# 1,25-Dihydroxyvitamin D_3_ regulates furin-mediated FGF23 cleavage

**DOI:** 10.1172/jci.insight.168957

**Published:** 2023-09-08

**Authors:** Han Xie, Isinsu Bastepe, Wen Zhou, Birol Ay, Zara Ceraj, Ignacio A. Portales-Castillo, Eva S. Liu, Sherri-Ann M. Burnett-Bowie, Harald Jüppner, Eugene P. Rhee, Murat Bastepe, Petra Simic

**Affiliations:** 1 Nephrology Division and; 2 Endocrine Division, Massachusetts General Hospital and Harvard Medical School, Boston, Massachusetts, USA.; 3 Endocrine Division, Brigham and Women’s Hospital and Harvard Medical School, Boston, Massachusetts, USA.; 4 Pediatric Nephrology Unit, Massachusetts General Hospital and Harvard Medical School, Boston, Massachusetts, USA.

**Keywords:** Endocrinology, Nephrology, Bone disease, Bone marrow

## Abstract

Intact fibroblast growth factor 23 (iFGF23) is a phosphaturic hormone that is cleaved by furin into N-terminal and C-terminal fragments. Several studies have implicated vitamin D in regulating furin in infections. Thus, we investigated the effect of 1,25-dihydroxyvitamin D_3_ [1,25(OH)_2_D] and the vitamin D receptor (VDR) on furin-mediated iFGF23 cleavage. Mice lacking VDR (*Vdr^–/–^*) had a 25-fold increase in iFGF23 cleavage, with increased furin levels and activity compared with wild-type (WT) littermates. Inhibition of furin activity blocked the increase in iFGF23 cleavage in *Vdr^–/–^* animals and in a *Vdr*-knockdown osteocyte OCY454 cell line. Chromatin immunoprecipitation revealed VDR binding to DNA upstream of the *Furin* gene, with more transcription in the absence of VDR. In WT mice, furin inhibition reduced iFGF23 cleavage, increased iFGF23, and reduced serum phosphate levels. Similarly, 1,25(OH)_2_D reduced furin activity, decreased iFGF23 cleavage, and increased total FGF23. In a post hoc analysis of a randomized clinical trial, we found that ergocalciferol treatment, which increased serum 1,25(OH)_2_D, significantly decreased serum furin activity and iFGF23 cleavage, compared with placebo. Thus, 1,25(OH)_2_D inhibits iFGF23 cleavage via VDR-mediated suppression of *Furin* expression, thereby providing a mechanism by which vitamin D can augment phosphaturic iFGF23 levels.

## Graphical abstract

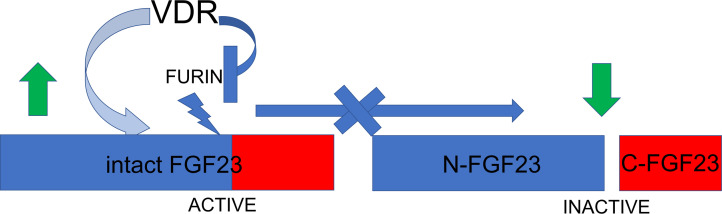

## Introduction

Fibroblast growth factor 23 (FGF23) is a phosphaturic hormone that is mainly produced by bone and bone marrow and thus plays a key role in phosphate homeostasis. Intact FGF23 (iFGF23) reduces serum phosphate levels by decreasing inorganic phosphate reabsorption in kidney proximal tubules and 1,25-dihydroxyvitamin D_3_ [1,25(OH)_2_D] synthesis (1). Inadequate iFGF23 production causes hyperphosphatemia and ectopic calcifications (2). Conversely, iFGF23 excess causes hypophosphatemia and skeletal mineralization defects (3). Elevated levels of FGF23 have been associated with an increased risk of cardiovascular events, such as heart failure, myocardial infarction, and cardiovascular mortality, with a potential causal role in promoting left ventricular hypertrophy, vascular calcification, and endothelial dysfunction (4, 5). Excess FGF23 also correlates with chronic kidney disease (CKD) and acute kidney injury, where it is associated with increased morbidity and mortality (6–8).

FGF23 is regulated at the transcriptional and posttranscriptional levels by several bone mineral factors, including phosphate (via glycerol-3-phosphate and lysophosphatidic acid) (9, 10), calcium, vitamin D, and parathyroid hormone (PTH) (11), and nonmineral factors like anemia, inflammation, and metabolism (12). Posttranscriptionally, FGF23 is regulated through cleavage. FGF23 comprises an N-terminal FGF homology domain and a C-terminal domain that interacts with the coreceptor Klotho. Full-length iFGF23 is required for the effects of FGF23 on kidney phosphate reabsorption and 1,25(OH)_2_D synthesis. Proteolytic degradation of iFGF23 into N-terminal and C-terminal cleavage fragments abrogates these biological effects. Further, the C-terminal FGF23 (cFGF23) fragment can block physiological FGF23 signaling by competing with iFGF23 for binding to the FGF receptor–Klotho complex (13). More recently, cFGF23 has been shown to directly upregulate iron levels during inflammation (14). It is therefore possible that cleavage products of FGF23 have important roles that are distinct from those of iFGF23, further emphasizing the impact of FGF23 cleavage regulation. FGF23 cleavage is increased during iron deficiency and inflammation (15) and in myelodysplastic syndrome (16). On the other hand, FGF23 cleavage is decreased in patients with CKD (17, 18) and during hyperphosphatemia (19). The degree of proteolytic cleavage can be assessed by quantitating the relative abundance of iFGF23 and cFGF23 cleavage fragments in circulation, utilizing ELISAs that specifically detect only iFGF23 or a combination of iFGF23 and cFGF23 fragments (20).

Proteases involved in cleaving FGF23 in osteoblasts in vitro include furin and proprotein convertase 5, but only furin was shown to mediate FGF23 cleavage in vivo (21). Furin is a calcium-dependent serine endoprotease that cleaves precursor proteins like prothrombin, proalbumin, proPTH, FGF23, and others (22). Dysregulated furin activity is associated with cancer, atherosclerosis, hypercholesterolemia, and infectious diseases caused by herpesvirus, coronavirus, or *Pseudomonas* (22). Furin cleaves iFGF23 at an Arg-XX-Arg site into N-terminal (aa 25–179) and C-terminal (aa 180–251) fragments (23). FGF23 is O-glycosylated at position 178 by N-acetylgalactosaminyltransferase 3 (GALNT3) (24), while the enzyme family with sequence similarity 20C (FAM20C) phosphorylates the hormone at position 180 (25). These modifications are regulated by phosphate and iron status, and are known to inhibit and promote furin-mediated FGF23 proteolysis, respectively (25–27). When furin is inactivated in osteoblasts and osteocytes in mice, circulating iFGF23 levels increase by 25% (21).

Several studies have proposed a putative role for vitamin D in regulating furin in the context of infections (28, 29). Gene set enrichment analysis of genomic data sets identified the vitamin D receptor (VDR) as being coexpressed with furin (30), which was the basis for the hypothesis that vitamin D might prevent SARS-CoV-2 infections by inactivating furin protease (28). Furthermore, furin inhibitors block SARS-CoV-2 spike protein cleavage, to suppress virus production and cytopathic effects in cell lines (31). So far, 11 randomized controlled trials investigated the therapeutic effects of vitamin D in COVID-19 with mixed results, with 4 trials reporting favorable effects and 7 trials reporting negative results (32).

1,25(OH)_2_D increases FGF23 levels in vivo and in vitro by upregulating *Fgf23* transcription (33–37). Conversely, VDR-knockout (*Vdr^–/–^*) mice have low serum FGF23 levels, which can be increased by feeding a rescue diet high in phosphate and calcium (38–41). However, the effect of 1,25(OH)_2_D on FGF23 cleavage has not been explored to our knowledge. In this study, using mouse models, cell-based assays, and data from a human trial, we investigated the effect of 1,25(OH)_2_D and the presence or absence of the VDR on furin-mediated cleavage of FGF23.

## Results

### FGF23 cleavage is increased in the absence of VDR.

**Figure 1:**
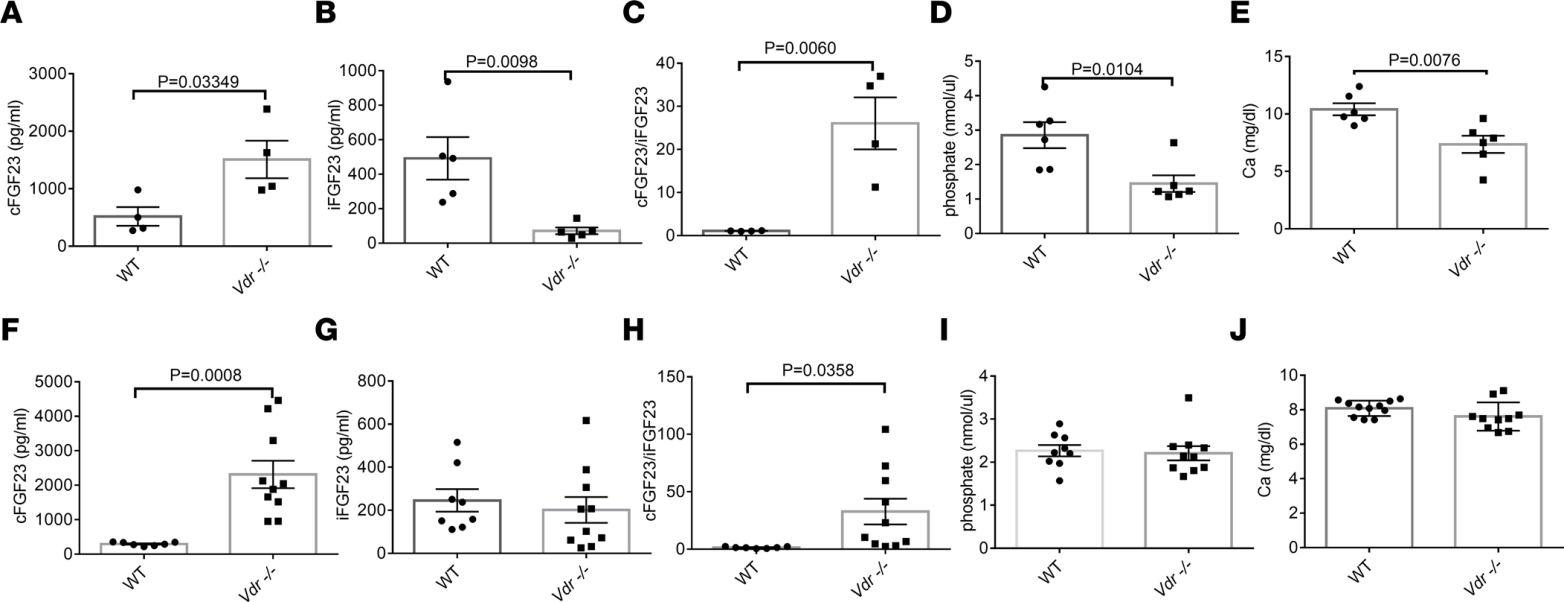
*Vdr^–/–^* mice display enhanced FGF23 cleavage. *Vdr^–/–^* mice at 7 weeks of age on regular diet have (**A**) higher cFGF23, (**B**) lower iFGF23, (**C**) higher cFGF23/iFGF23, and lower (**D**) phosphate and (**E**) calcium levels than WT controls (*n* = 4–5 per group). *Vdr^–/–^* mice at 9 weeks of age on rescue diet for 2 weeks have (**F**) higher cFGF23, (**G**) no change in iFGF23, (**H**) higher cFGF23/iFGF23, and similar (**I**) phosphate and (**J**) calcium levels as compared to WT mice (*n* = 6 per group). Data represent the mean ± SEM. *P* values by 2-tailed Student’s *t* test.

Compared with wild-type (WT) littermates, *Vdr^–/–^* mice at 7 weeks of age on a normal diet had more cFGF23 and less iFGF23, with a 25-fold increase in cleavage of iFGF23 (cFGF23/iFGF23) (Figure 1, A–C). In the absence of *Vdr*, there was a reduction in both phosphate and calcium (Figure 1, D and E) with elevated PTH (Supplemental Figure 1A; supplemental material available online with this article; https://doi.org/10.1172/jci.insight.168957DS1). To exclude an effect of low phosphate and calcium on FGF23 cleavage, we fed *Vdr^–/–^* mice a high-phosphate and -calcium diet (1.25% and 2.0%, respectively) and showed that these animals continued to have increased cFGF23 despite the normalization of iFGF23, with a persistent increase in FGF23 cleavage (cFGF23/iFGF23) compared with WT mice (Figure 1, F–H). The total amount of FGF23 was increased by the rescue diet as previously described (39) because of the stimulatory effect of both phosphate and calcium on FGF23 synthesis that is independent of calcitriol (39, 42, 43). The rescue diet normalized serum phosphate, calcium, and PTH levels of *Vdr^–/–^* to comparable levels relative to WT mice (Figure 1, I and J, and Supplemental Figure 1B). Thus, we further focused on *Vdr^–/–^* mice receiving the rescue diet.

### Vdr^–/–^ mice have more Furin mRNA, protein, and activity.

**Figure 2:**
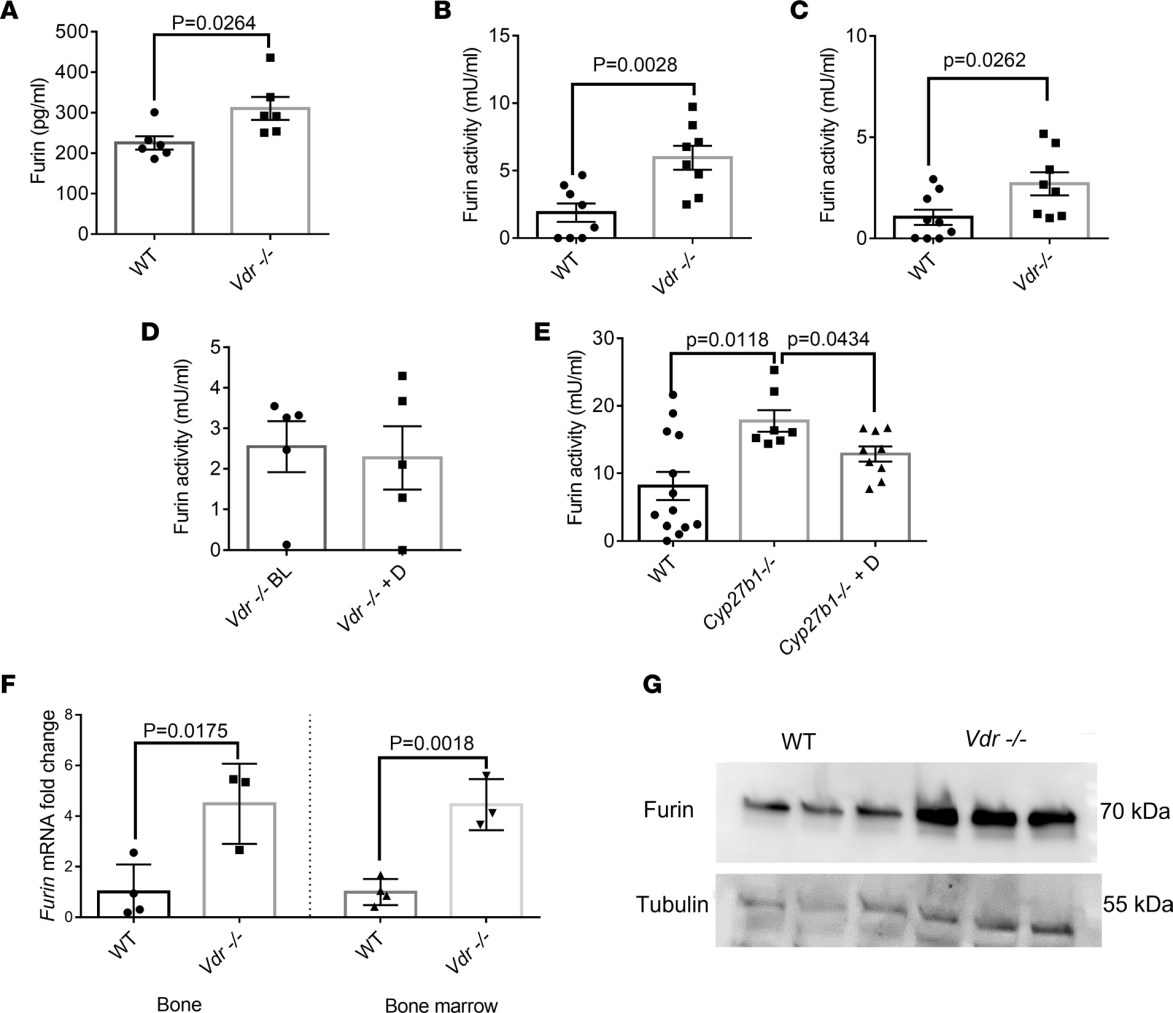
*Vdr^–/–^* mice have higher furin levels and activity than WT mice. *Vdr^–/–^* mice have an increase in furin (**A**) protein (*n* = 6 per group) and activity in plasma than mice on rescue diet (**B**) and normal diet (*n* = 8 per group) (**C**). The effect of calcitriol treatment on furin activity in *Vdr^–/–^* (*n* = 5 per group) (**D**) and *Cyp27b1^–/–^* mice (*n* = 7–12 per group) (**E**). *Furin* mRNA in bone and bone marrow (*n* = 3–4 per group) (**F**) and furin protein in bone marrow (**G**). qRT-PCR was performed using *Gapdh* as the relative control, and data were subsequently normalized to the levels in WT. Tubulin was used as a loading control in Western blots. Data represent the mean ± SEM. Data represent the mean ± SEM. *P* values by 2-tailed Student’s *t* test (**A**–**D** and **F**) or 2-tailed Student’s *t* test with Bonferroni’s correction (**E**). BL, baseline; D, calcitriol; qRT, quantitative reverse transcription.

Furin is the main protease involved in the cleavage of iFGF23. Because *Vdr^–/–^* mice have increased cleavage of iFGF23, we investigated the effect of *Vdr* ablation on furin. We first showed that *Vdr^–/–^* mice had significantly increased furin protein levels and activity in the serum (Figure 2, A and B). Furin activity was also increased in *Vdr^–/–^* mice on the nonrescue diet (Figure 2C). 1,25(OH)_2_D treatment failed to reduce furin activity and to normalize cleavage of FGF23 in *Vdr^–/–^* mice, suggesting that the effect of 1,25(OH)_2_D on furin cleavage depends on the VDR and is not mediated through nongenomic effects of this biologically active vitamin D (Figure 2D and Supplemental Figure 2, A–E). By contrast, *Cyp27b1^–/–^* mice, i.e., animals with a global ablation of 25-hydroxyvitamin D-1α-hydroxylase that converts inactive 25-OHD into biologically active 1,25(OH)_2_D, had increased furin activity at baseline that was reduced by 1,25(OH)_2_D treatment (Figure 2E). Next, we measured *Furin* gene expression at the site of FGF23 production in the bone and bone marrow and showed a nearly 5-fold increase in *Furin* mRNA in *Vdr^–/–^* mice (Figure 2F). Subsequently, we detected an increase in the level of furin protein in the bone marrow in the absence of VDR (Figure 2G). There was no difference between WT and *Vdr*^–/–^ mice in anemia or inflammatory parameters that increase FGF23 cleavage, as measured by hemoglobin, iron levels, transferrin saturation, and IL-1β levels (Supplemental Figure 3, A–D). Klotho levels in the systemic circulation and locally in the bone marrow were similar between *Vdr^–/–^* and WT mice (Supplemental Figure 4, A and B).

### Furin inhibition attenuates the enhanced cleavage of FGF23 in Vdr^–/–^ mice and in Vdr-knockdown cells and reduces cleavage of FGF23 and serum phosphate in WT mice.

**Figure 3:**
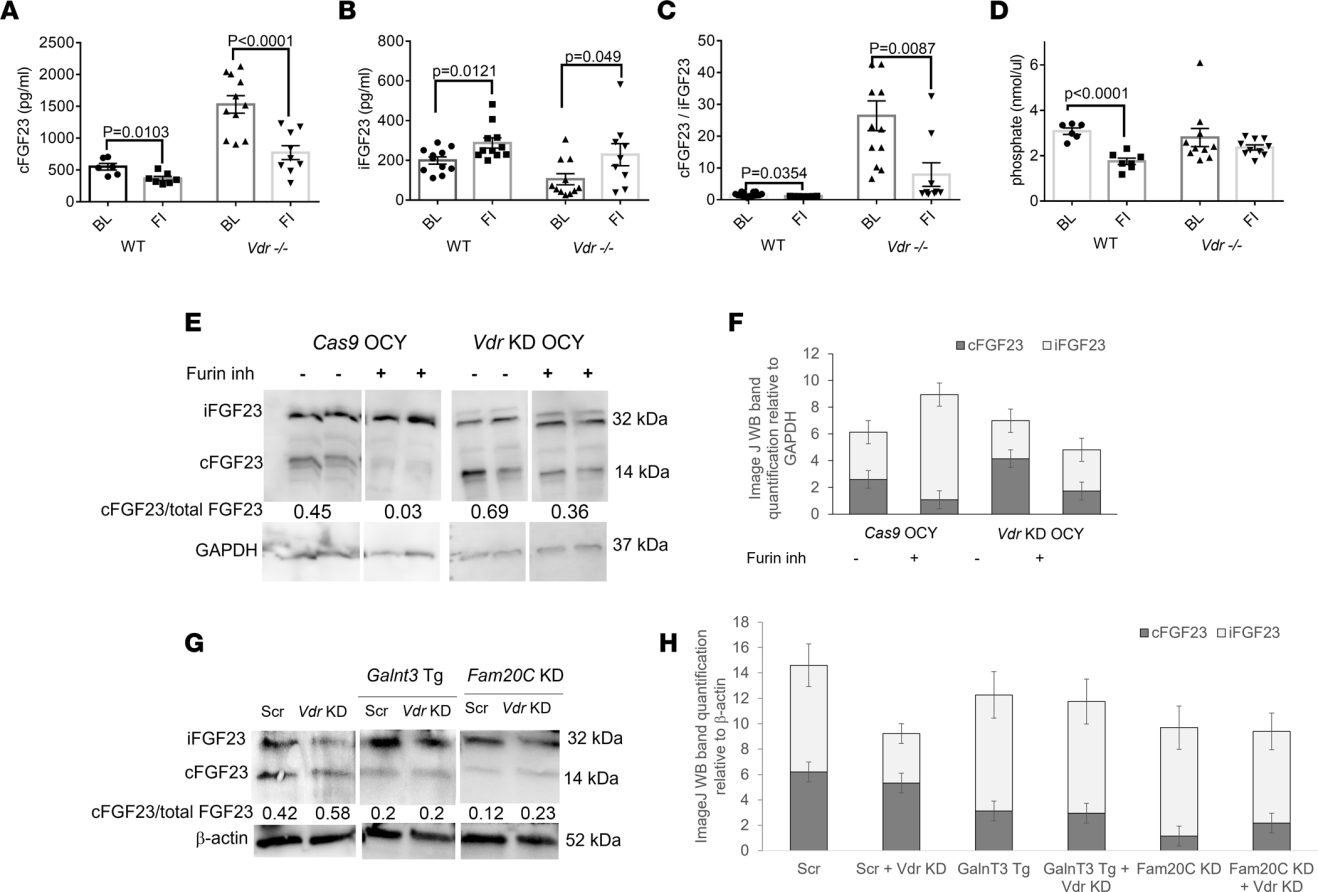
Furin inhibition reduces FGF23 cleavage in vivo and in vitro. Treatment with a furin inhibitor (**A**) reduces cFGF23, (**B**) increases iFGF23, and (**C**) reduces cFGF23/iFGF23 in the plasma of WT and *Vdr^–/–^* mice (*n* = 6–11 per group). (**D**) The furin inhibitor diminishes serum phosphate in WT mice. (**E**) OCY454 cells with a knockdown of *Vdr* (*Vdr*-KD OCY) exhibit a higher cFGF23/iFGF23 ratio than control cells (*Cas9* OCY), and furin inhibition increases the relative abundance of iFGF23 in both *Cas9* OCY and *Vdr* KD OCY cells. (**F**) Quantification of Western blot bands by ImageJ. (**G**) *Vdr* KD does not increase cFGF23 in OCY454 with *GalnT3* overexpression (*GalnT3* Tg) and *Fam20C* KD; β-actin blots are derived from the same samples run contemporaneously in parallel gels. (**H**) Quantification of Western blot bands by ImageJ (NIH). Data represent the mean ± SEM. *P* values by 2-tailed Student’s test. FI, furin inhibitor; Scr, scrambled.

As *Vdr^–/–^* mice have both increased furin activity/levels and iFGF23 cleavage, we used Decanoyl-RVKR-CMK, a furin inhibitor, to test if furin in *Vdr^–/–^* mice is responsible for the increase in FGF23 cleavage. The furin inhibitor reduced FGF23 cleavage in *Vdr^–/–^* mice within 7 days, as shown by the decrease in cFGF23 and cFGF23/iFGF23 ratio in *Vdr^–/–^* mice treated with the furin inhibitor, to levels comparable to those encountered in WT mice (Figure 3, A–C). Furthermore, in WT mice, the furin inhibitor suppressed the level of cFGF23 and elevated the level of iFGF23 in serum, decreasing cFGF23/iFGF23 ratio (Figure 3, A–C). Consequently, WT mice treated with the furin inhibitor had a reduction in serum phosphate (Figure 3D). There were no significant changes in PTH and calcium in response to the furin inhibitor (Supplemental Figure 5, A and B).

To exclude the effect of systemic factors, we tested the effect of VDR and the furin inhibitor in OCY454 cells, an osteocyte-like cell line (44). CRISPR/Cas9-mediated knockdown of *Vdr* (*Vdr* KD, Supplemental Figure 6) led to an increase in the abundance of cFGF23 and a decrease in iFGF23 compared with Cas9-expressing control OCY454 cells, suggesting there was more cleavage of FGF23 (Figure 3, E and F). Administration of the furin inhibitor reduced the cleavage of FGF23 in Cas9-expressing cells and *Vdr*-KD cells, as judged by decreased cFGF23 and increased iFGF23 levels in cell lysates (Figure 3, E and F).

Next, we investigated the interaction between VDR and 2 established molecular regulators of FGF23 cleavage, GALNT3 and FAM20C. GALNT3 is responsible for glycosylating FGF23, a process that impedes its cleavage, while FAM20C phosphorylates FGF23, making it less susceptible to glycosylation and more prone to cleavage. There was no difference in *Galnt3* and *Fam20C* mRNA expression in the bone marrow of *Vdr*^–/–^ mice versus WT mice in vivo (Supplemental Figure 7, A and B). However, the KD of *Vdr* in the OCY454 cell line increased protein levels of GALNT3 and reduced levels of FAM20C (Supplemental Figure 7, C and D). These changes would tend to reduce FGF23 cleavage, suggesting that the increase in FGF23 cleavage observed following *Vdr* KD is not mediated through GALNT3 or FAM20C. Further, overexpression of *Galnt3* and KD of *Fam20C* (Supplemental Figure 8, A and B) in OCY454 cells blocked the effect of *Vdr* KD on increased FGF23 cleavage (Figure 3, G and H), consistent with their effect on FGF23 cleavage at the furin cleavage site, downstream of the *Vdr*-mediated impact on furin.

### VDR suppresses Furin transcription.

**Figure 4:**
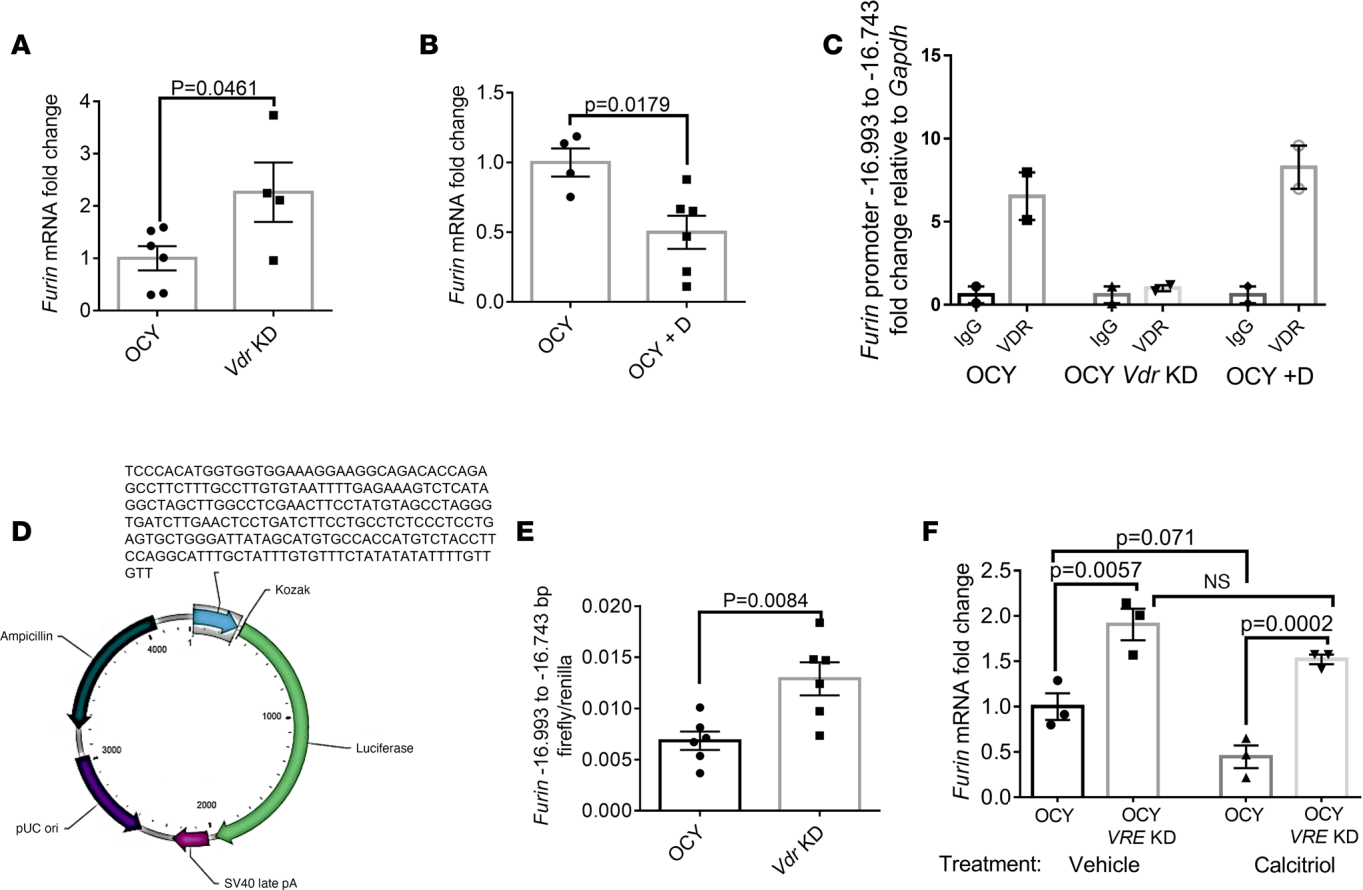
VDR suppresses *Furin* transcription. (**A**) *Vdr*-KD OCY cells have more *Furin* mRNA transcription than control cells (*n* = 4–6 per group), while (**B**) calcitriol reduces *Furin* mRNA (qRT-PCR was performed relative to *Gapdh*, and data were subsequently normalized to control cells) (*n* = 4–6 per group). (**C**) ChIP of the *Furin* promoter in WT, *Vdr*-KD OCY, and OCY cells treated with calcitriol. Plot shows relative enrichment of the putative VDR target site pulled down by an antibody against VDR compared with IgG. (**D**) Plasmid scheme of furin DNA, universal promotor, and luciferase reporter. (**E**) Firefly luciferase/renilla luciferase activity showing more *Furin* –16,993 bp to –16,743 bp–regulated promoter activity in *Vdr*-KD OCY cells (*n* = 6 per group). (**F**) *Furin* mRNA transcription in OCY cells with *VRE* KD versus controls treated with vehicle and calcitriol (*n* = 3 per group). Data represent the mean ± SEM. *P* values by 2-tailed Student’s *t* test (**A**, **B**, and **E**) or 2-way ANOVA (**F**). D, calcitriol; VRE, vitamin D responsive element.

We next investigated a plausible interaction between VDR and *Furin* in OCY454 cells. We first recapitulated *Furin* expression results from in vivo models in the osteocyte cell line, showing that *Vdr*-KD OCY454 cells had more *Furin* expression (Figure 4A). Conversely, 1,25(OH)_2_D treatment reduced *Furin* mRNA expression in OCY454 cells (Figure 4B). We used the JASPAR CORE 2022 program (https://jaspar.genereg.net) to predict VDR binding sites upstream of the *Furin* gene. Chromatin immunoprecipitation (ChIP) experiments showed enrichment of VDR at the putative VDR binding site located –16,993 bp to –16,743 bp of the *Furin* gene (Figure 4C and Supplemental Figure 9, A–D). We then subcloned the identified *Furin* DNA region (VDR responsive element, VRE) with a universal promoter and firefly luciferase (Figure 4D). This construct displayed increased promoter activity in *Vdr*-KD cells compared with control OCY454 cells (Figure 4E and Supplemental Figure 9E). Similarly, CRISPR KD of the *VRE* in the *Furin* promoter led to an increase in *Furin* transcription that was not suppressed by 1,25(OH)_2_D treatment (Figure 4F and Supplemental Figure 10). Together, these findings show that the –16,993 bp to –16,743 bp *VRE* suppresses *Furin* transcription.

### 1,25(OH)_2_D reduces FGF23 cleavage and furin activity in vivo.

**Figure 5:**
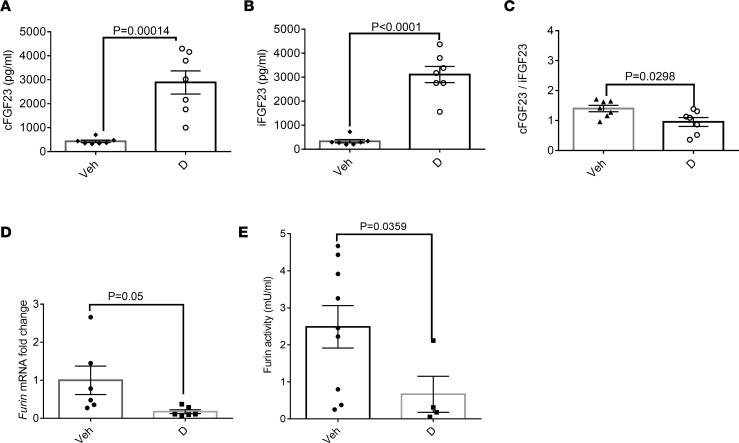
1,25(OH)_2_D reduces cleavage of FGF23 and furin activity in WT mice. 1,25(OH)_2_D (D) 0.5 μg every other day i.p. for 7 days increases both (**A**) cFGF23 and (**B**) iFGF23 but (**C**) reduces cFGF23/iFGF23 (*n* = 7 per group). Remainder of bone mineral metabolism analyses are in Supplemental Figure 11, A–C. (**D**) Calcitriol reduces *Furin* mRNA in the bone marrow relative to *Gapdh* (*n* = 6 per group) and (**E**) reduces furin activity in plasma in WT mice (*n* = 4–9 per group). Data represent the mean ± SEM. *P* values by 2-tailed Student’s *t* test.

To test the effect of 1,25(OH)_2_D directly in vivo, we injected WT mice with calcitriol. This treatment increased total FGF23 (cFGF23 and iFGF23), while it reduced the cFGF23/iFGF23 ratio (Figure 5, A–C). Animals treated with 1,25(OH)_2_D displayed a reduction of *Furin* transcription in the bone marrow and furin activity in plasma (Figure 5, D and E). As expected, the treatment increased serum calcium and reduced PTH levels, and these changes were associated with modestly reduced serum phosphate levels (Supplemental Figure 11).

As FGF23 cleavage is decreased in patients with CKD, we tested if there was a role for 1,25(OH)_2_D or *Vdr*^–/–^ in modulating cleavage of FGF23 in a CKD mouse model. WT and *Vdr^–/–^* mice were fed an adenine-rich diet for 4 weeks, which induced CKD (45), as verified by a rise in serum creatinine (Supplemental Figure 12A). CKD resulted in reduced FGF23 cleavage, which was exacerbated by administration of 1,25(OH)_2_D (Supplemental Figure 12, B–D). However, CKD significantly reduced FGF23 cleavage in *Vdr*^–/–^ mice (Supplemental Figure 12E), suggesting that the effect of *Vdr* deficiency on cleavage is counteracted by other factors in CKD.

### 1,25(OH)_2_D reduces furin activity and FGF23 cleavage in humans.

**Figure 6:**
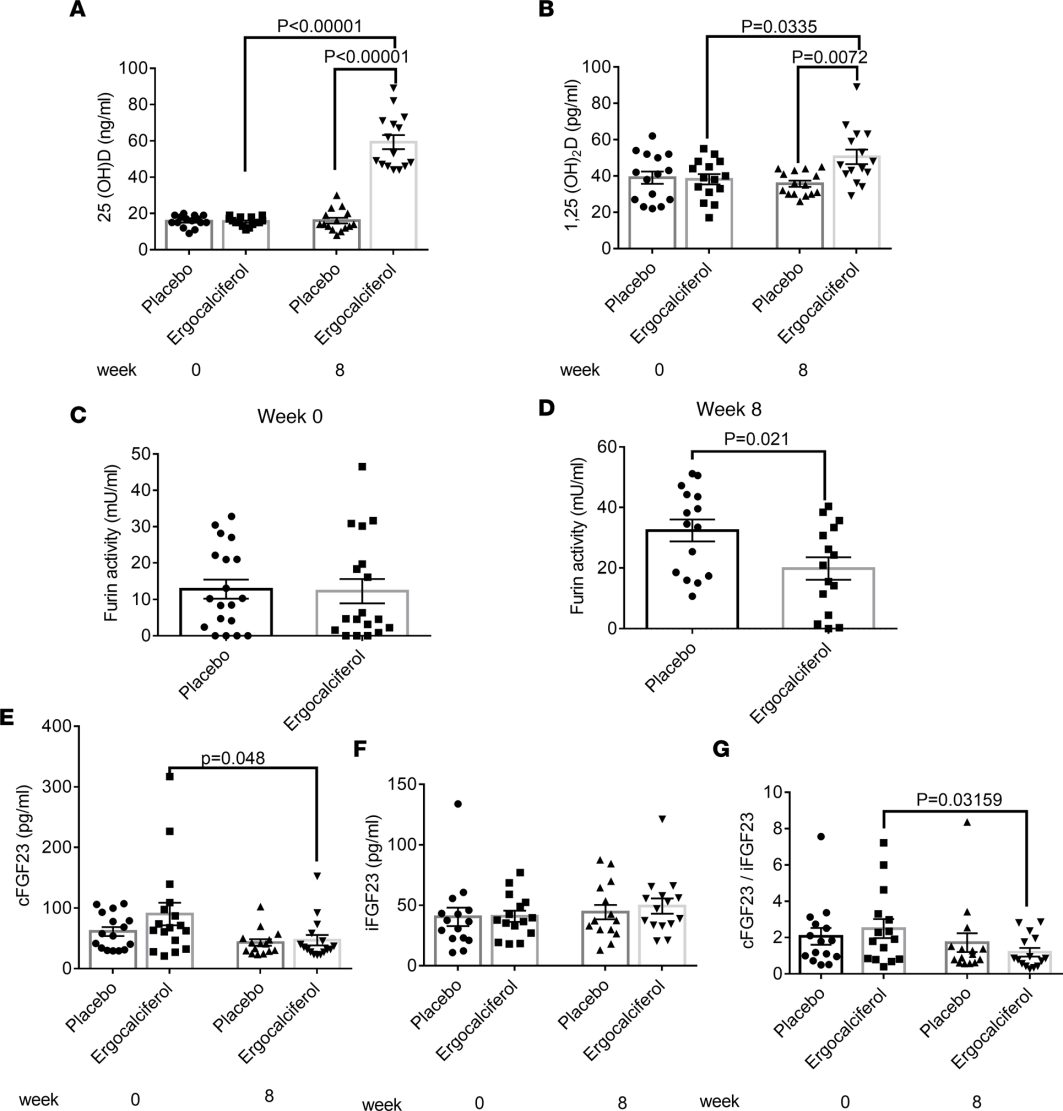
Ergocalciferol treatment reduces furin activity in patients. Ergocalciferol 50,000 IU orally once a week increases (**A**) 25(OH) vitamin D and (**B**) 1,25(OH)_2_ vitamin D as compared with placebo treatment in patients with vitamin D deficiency. (**C**) There is no difference in furin activity at baseline in patients with vitamin D deficiency. (**D**) At 8 weeks of treatment, furin activity is decreased in patients treated with ergocalciferol as compared with placebo-treated patients. (**E**) Ergocalciferol reduces cFGF23 after 8 weeks of treatment as compared with baseline. (**F**) There is no difference in iFGF23 between the treatment and placebo groups. (**G**) Ergocalciferol reduces cFGF23/iFGF23 after 8 weeks of treatment as compared with baseline. *n* = 18 per group. Data represent the mean ± SEM. *P* values by 2-way ANOVA test (**A** and **B**) or 2-tailed Student’s *t* test (**C**–**G**).

We analyzed a subset of samples from a randomized trial assessing the effects of ergocalciferol administration on circulating FGF23 (46). We randomized 18- to 45-year-old individuals with 25-OHD levels ≤ 20 ng/mL to weekly ergocalciferol treatment of 50,000 IU or placebo, consuming a self-selected diet. There was no difference in bone mineral parameters and kidney function at baseline (Supplemental Table 1). We tested the effect of ergocalciferol on furin activity after 8 weeks of treatment, since iFGF23 levels had been maximally increased at that time point (46). As expected, in comparison with the placebo-treated group, serum 25-OHD and 1,25(OH)_2_D levels were significantly increased in the ergocalciferol-treated group (Figure 6, A and B). Furin activity was significantly decreased in patients receiving ergocalciferol versus placebo (Figure 6, C and D). Furthermore, we showed that ergocalciferol treatment reduced FGF23 cleavage, as shown by the reduction of cFGF23 and cFGF23/iFGF23 ratio in individuals receiving ergocalciferol (Figure 6, E–G). Consistent with the original study, a trend toward increased iFGF23 levels in response to ergocalciferol treatment was observed (Figure 6F).

## Discussion

**Figure 7:**
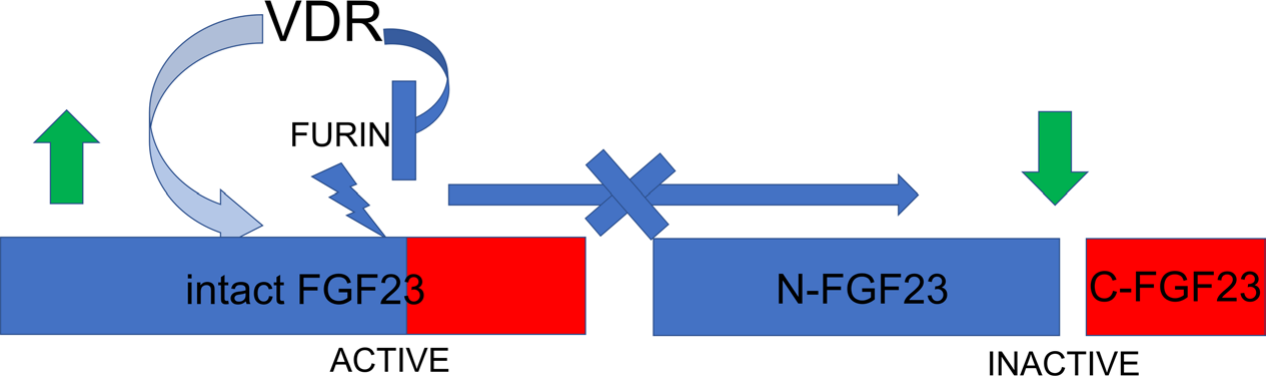
Schema depicting the action of VDR on suppressing furin and FGF23 cleavage. VDR suppresses *Furin* transcription and activity, thus reducing the cleavage of intact FGF23 into N-terminal FGF23 (N-FGF23) and cFGF23 (C-FGF23). Hence, the level of biologically active intact FGF23 increases upon VDR action.

Our study reveals that VDR deficiency results in enhanced FGF23 cleavage that is dependent on increased furin activity. Blocking furin in *Vdr^–/–^* animals fully restores FGF23 cleavage to baseline levels. Conversely, exogenous 1,25(OH)_2_D reduces FGF23 cleavage and furin activity (Figure 7).

VDR deficiency leads to PTH elevation, and PTH injections increase furin and cFGF23 levels in mice (47). Due to this possible confounding effect of PTH, we suppressed PTH levels with a high-calcium and -phosphate diet (rescue diet) in *Vdr^–/–^* mice. Moreover, the effect of 1,25(OH)_2_D injection on cFGF23/iFGF23 levels in mice persisted for at least 48 hours postinjection, while it has been shown that the effect of PTH on cFGF23/iFGF23 resolves within 4 hours of injection (47). Thus, the effects of 1,25(OH)_2_D on FGF23 cleavage observed in our in vivo experiments are unlikely to be dependent on the alterations in PTH levels. Additionally, our in vitro results, which are not affected by PTH alterations, further support a direct effect of VDR on *Furin*. Indeed, our results suggest that VDR binds to a long-range regulatory element of the *Furin* gene at about 16 kb prior to the *Furin* transcription start site. Such an action of VDR is not unusual. Combining the microarray data of 1,25(OH)_2_D treatments with VDR ChIP-Seq in monocytes revealed that most VDR binding sites are within 400 kb of the transcription start site, and in the case of downregulated genes, 57% of those sites were even further upstream (48).

GALNT3 glycosylates FGF23 at amino acid residue T178, located within the furin cleavage site, thus preventing its proteolytic processing and enabling the secretion of iFGF23 (49). In contrast, FAM20C phosphorylates residue S180 in the same FGF23 region, thereby inhibiting GALNT3-mediated O-glycosylation and promoting FGF23 cleavage by furin (25). VDR does not mediate its effect on FGF23 cleavage through GALNT3 or FAM20C, as the observed changes in the levels of these proteins in VDR-deficient cells are predicted to reduce FGF23 cleavage. Further, *Galnt3* overexpression and *Fam20C* KD abrogated the increase in FGF23 cleavage observed in *Vdr*-KD cells. Since both GALNT3 and FAM20C regulate FGF23 susceptibility to furin cleavage, VDR, which controls *Furin* expression, cannot override the effects of GALNT3 and Fam20C. Consequently, genetic disorders caused by inactivating *GalnT3* and *Fam20C* mutations, such as familial tumoral calcinosis (49) and hypophosphatemic rickets (50), are unlikely to be influenced by VDR-mediated regulation of FGF23 cleavage.

Erythropoietin has been found to decrease *GalnT3* mRNA levels without affecting *Furin* mRNA levels (51). This reduction in *GalnT3* mRNA may contribute to an increase in FGF23 cleavage under conditions characterized by elevated erythropoietin, such as iron deficiency anemia, inflammation, or exogenous treatment with synthetic erythropoietin. As a result, it is plausible that the influence of 1,25(OH)_2_D on FGF23 may be diminished under these circumstances. However, the overall impact would depend on the extent of the reduction in GALNT3 relative to furin. On the other hand, high-phosphate diet enhances the skeletal expression of *Galnt3*, with expected increases in serum iFGF23 (19, 25). Increase in iFGF23 leads to reduced production of 1,25(OH)_2_D in the kidney, which would in turn increase iFGF23 cleavage, serving as a negative feedback loop. Additional experiments are required to gain a clearer understanding of the interactions between different mechanisms involved in FGF23 cleavage and vitamin D metabolism.

As VDR deficiency and 1,25(OH)_2_D directly affect the transcription of many hormones, especially ones involved in mineral metabolism like FGF23 and PTH, the end effect on phosphate is difficult to interpret. The VDR-regulated furin system that we outline seems to be physiologically relevant, as serum phosphate concentration was reduced in WT mice treated with a furin inhibitor, a finding expected with reduction of FGF23 cleavage. However, we were not able to demonstrate an effect of the furin inhibitor on serum phosphate in *Vdr^–/–^* mice, likely owing to our repleting calcium and phosphate to dampen PTH. The VDR-regulated furin system we outline is also observed in humans, as we found that ergocalciferol treatment in patients with vitamin D deficiency reduces furin activity and FGF23 cleavage. For the subset of samples analyzed in the current study (*n* = 36), there was only a trend toward increased iFGF23 levels in response to ergocalciferol. However, this is attributable to the small sample size, as ergocalciferol treatment did result in a significant increase in iFGF23 in the complete study sample (*n* = 90) (46).

CKD increases FGF23 production, but reduces cleavage, resulting in an overall predominance of iFGF23 (18, 52). This is true even when there is concurrent iron deficiency or inflammation, factors that usually increase FGF23 cleavage and are highly prevalent in CKD. Similarly, whereas vitamin D deficiency is prevalent in CKD, our findings demonstrate that the influence of CKD on FGF23 cleavage is dominant over the effects of *Vdr*^–/–^, an extreme in vivo model of severe vitamin D deficiency. Nevertheless, treatment with vitamin D does appear to further reduce FGF23 cleavage even in the context of CKD, consistent with human data showing increased iFGF23 levels after calcitriol treatment in patients with kidney injury (53). Therefore, it is interesting to consider that the vitamin D deficiency in CKD may have some compensatory effect toward reducing iFGF23 levels.

Although we focused on FGF23, vitamin D might also play a role in furin-regulated cleavage of other prohormones. In particular, 1,25(OH)_2_D may impair furin-mediated cleavage of proPTH, since it has been shown that furin in parathyroid glands cleaves proPTH into the 6– amino acid “pro” sequence and the biologically active, secreted PTH(1-84) (54). Accordingly, we detected a trend toward a decrease in circulating PTH levels after inhibiting furin activity in *Vdr*^–/–^ mice. Without the rescue diet, these mice have markedly increased PTH levels, which is probably related to reduced intestinal calcium absorption and thus decreased blood calcium levels. Furthermore, elevated PTH levels likely reflect reduced 1,25(OH)_2_D action directly on the parathyroid gland and possibly increased cleavage of proPTH. It could perhaps be speculated that 1,25(OH)_2_D may simultaneously reduce the cleavage of FGF23 (resulting in more biologically active iFGF23) and proPTH (resulting in less biologically active PTH) to mitigate potential increases in blood phosphate and calcium. From a broader perspective, it is important to note that furin targets many other proteins. Hence, the importance of this mechanism may extend beyond the phosphate metabolism and calciotropic hormones and could establish a role for vitamin D treatment in furin-related disorders, like infectious diseases or cancer (22).

It is interesting to speculate why 1,25(OH)_2_D might augment overall FGF23 bioactivity through both an effect on FGF23 transcription and a posttranscriptional cleavage mechanism. Unlike iron deficiency and inflammation, where increased FGF23 production is coupled to increased cleavage (15), 1,25(OH)_2_D uncouples both mechanisms, thus leading to a net increase in iFGF23. As noted, this ensures that activated vitamin D analogs enhance the production of biologically active FGF23, which can help mitigate intestinal 1,25(OH)_2_D-induced phosphate absorption. As new insights into the functions of cFGF23 continue to emerge, the regulatory mechanisms of FGF23 cleavage may have broader implications. For instance, cFGF23 has been found to elevate iron levels during inflammation (14) and counteract the effects of iFGF23 (13, 19). The significant 25-fold increase in cFGF23 observed in *Vdr*^–/–^ mice may have physiological implications beyond the mere reduction of iFGF23 and its effect on phosphate.

Overall, our study reveals a physiology of vitamin D and VDR suppressing furin and FGF23 cleavage. This mechanism that augments the biological activity of FGF23 warrants further exploration in different FGF23-related conditions such as hyperphosphatemia, CKD, anemia, or inflammation.

## Methods

### Mice.

All studies were approved by the IACUC of Massachusetts General Hospital. Age- and sex-matched C57BL/6J *Vdr^–/–^* mice (The Jackson Laboratory, RRID:IMSR_JAX:006133) (55), *Cyp27b1^–/–^* mice (a gift from Rene St-Arnaud, Genetics Unit, Shriners Hospital for Children, Montreal, Quebec, Canada) (56), and their WT controls were maintained in a specific pathogen–free environment at 25°C on a 12-hour light/12-hour dark cycle. WT and *Vdr^–/–^* mice were fed the standard diet or a rescue diet (TD96348, Teklad) containing 2% calcium, 1.25% phosphorus, and 20% lactose supplemented with 2.2 IU vitamin D/g. The rescue diet has been shown to prevent abnormalities in mineral ion levels and the consequent development of hyperparathyroidism and osteomalacia/rickets in *Vdr*^–/–^ mice (57, 58).

### Animal experiments.

1,25(OH)_2_D (0.5 mg i.p., Enzo Life Sciences) and furin inhibitor Decanoyl-RVKR-CMK (0.14 mg/mouse i.p., MilliporeSigma) were injected every other day for a total of 7 days. The mice were sacrificed after 7 days of treatment, and blood, femurs, and bone marrow were collected for further analysis. *Cyp27b1^–/–^* mice were treated with 1,25(OH)_2_D 175 pg/g/d for 30 days, and plasma was collected for further analysis. WT and *Vdr*^–/–^ mice, at 8 weeks of age, were given adenine-rich chow diet (0.2% adenine) for a total of 4 weeks (45). WT mice on adenine-rich diet were randomly divided into animals receiving 1,25(OH)_2_D (0.5 mg i.p.) or vehicle. Blood was collected every 2 weeks.

### Mouse plasma measurements.

All plasma iFGF23, cFGF23, and PTH levels were measured by the respective Immutopics ELISAs according to the manufacturer’s instructions. Phosphate was measured by colorimetric assay (Abcam). Calcium was measured by Calcium CPC LiquiColor Test kits (Stanbio Laboratory). Furin was measured by ELISA (Biomatik) and furin activity by the BioVision kit from plasma according to manufacturers’ instructions. Klotho protein was measured by Immunotag ELISA, hemoglobin by Massachusetts General Hospital Center for Comparative Medicine Core Laboratory, iron and transferrin saturation by BioVision kit, and IL-1β by Invitrogen (Thermo Fisher Scientific) ELISA.

### Cell culture and experiments.

OCY454 cells were grown from a single subclone (Center for Skeletal Research, Massachusetts General Hospital) (44, 59). Cells were passaged in α-MEM supplemented with 10% heat-inactivated FBS and 1% antibiotics (penicillin-streptomycin; Fungizone) at 33°C in 5% CO_2_. Cells were plated at 50,000 cells/mL and allowed to reach confluence at 33°C (typically in 2–3 days) prior to transfer to a 37°C environment. Experiments were performed on cells cultured at 37°C for 7 days. OCY454 cells, with or without gene deletions, were treated with Decanoyl-RVKR-CMK 50 μM for 48 hours or calcitriol 10 nM for 24 hours. UMR106 cells (ATCC, CRL-1661) were cultured at 37°C in DMEM supplemented with 10% FBS and 1% antibiotics.

### Vdr and Vre deletion.

CRISPR/Cas9-mediated gene deletions were performed using the sgRNA targeting sequences listed in Supplemental Table 1. First, OCY454 cells were stably transduced with a blasticidin resistance–conferring Cas9-expressing lentivirus. sgRNA sequences were subcloned into lentiGuide-Puro (Addgene plasmid 52963) (9, 60). OCY454 cells were transfected with this plasmid using Fugene HD (Promega; 1 μg plasmid per well of a 6-well plate). Lentivirus particles were produced in HEK293T cells (ATCC, CRL-3216) using standard protocols (Broad Institute, http://www.broadinstitute.org/rnai/public/resources/protocols). Cells were exposed to lentivirus particles (MOI = 1) overnight at 33°C in the presence of polybrene (5 μg/mL). Media were then changed, and puromycin (2 μg/mL) and blasticidin (4 μg/mL) were added. Cells were maintained in selection medium throughout the duration of the experiment. shRNA transient KD was performed by shRNA lentivirus particles for *Vdr* that were purchased from Origene (catalog TG513148) and were transfected in UMR106 cells (ATCC, CRL-1661) using Lipofectamine 2000 (Thermo Fisher Scientific) per manufacturer’s instructions.

### Galnt3 overexpression and Fam20C KD.

Lentiviral ORF cDNA for *Galnt3* or shRNA lentivirus particles for *Fam20C* were purchased from Origene (catalogs MR223895L4V and TL515201V). Viral packaging was performed in HEK293T cells using standard protocols (Broad Institute, http://www.broadinstitute.org/rnai/public/resources/protocols). OCY454 cells were exposed to lentivirus particles (MOI = 1) overnight in the presence of polybrene (5 μg/mL). Cells were subsequently selected with puromycin (2 μg/mL) and maintained in selection medium throughout the duration of the experiment.

### qRT-PCR.

Total RNA was extracted from cultured cells using RNeasy (QIAGEN) following the kit manufacturer’s instructions. For long bone RNA isolation, mice were euthanized, and femurs were rapidly dissected on ice. Soft tissue was removed, and epiphyses were cut. Bone marrow cells were then removed by serial flushing with ice-cold PBS. TRIzol (Life Technologies, Thermo Fisher Scientific) was added, and samples were frozen at –80°C and then homogenized. For bone marrow RNA, flushed bone marrow was spun down and homogenized with TRIzol. RNA was then extracted according to the manufacturer’s instructions. For cDNA synthesis, 1 μg RNA was used for the synthesis reactions according to the manufacturer’s instructions (PrimeScript RT; Takara). SYBR Green–based qRT-PCR detection was performed using FastStart Universal SYBR Green (Roche) on a StepOne Plus Thermocycler (Applied Biosystems, Thermo Fisher Scientific). The PCR primer sequences are listed in Supplemental Table 1.

### Immunoblotting.

Whole-cell lysates were prepared using RIPA buffer with protease inhibitors (Pierce, Thermo Fisher Scientific). Bone marrow from femurs and tibiae of C57BL/6J mice was flushed in RIPA buffer with protease inhibitors. The samples were centrifuged at 13,000*g* for 10 minutes at 4°C, and the supernatants were collected. Lysates (15–20 μg cellular protein) were separated by SDS-PAGE, and proteins were transferred onto nitrocellulose membranes. Membranes were blocked with 5% milk in TBS-Tween and incubated with FGF23 (186-206, 21-6310; Immutopics), VDR (ab109234, Abcam), Furin (sc-133142, Santa Cruz Biotechnology), GALNT3 (PA5-89560, Invitrogen, Thermo Fisher Scientific) or FAM20C (25395-1-AP, Proteintech) antibodies overnight at 4°C. The next day, the membranes were washed and incubated with the appropriate HRP-coupled secondary antibodies (anti-mouse, 62-6520; anti-rabbit, G-21234, anti-goat, A15999 antibodies, Invitrogen, Thermo Fisher Scientific), and signals were detected with ECL (Pierce, Thermo Fisher Scientific). All immunoblots were repeated at least twice, with comparable results.

### ChIP analysis.

ChIP analysis of 107 OCY454 cells was performed with a Magna ChIP Kit (MilliporeSigma) using a VDR antibody (ab8756; Abcam) and IgG (sc-2025; Santa Cruz Biotechnology). The primer sequences are listed in Supplemental Table 1. qRT-PCR was performed to amplify immunoprecipitated genomic fragments, and data are presented as the fold-change in enrichment relative to IgG-associated DNA.

### Luciferase assay.

Transcription of *Furin* –16,993 bp to –16,743 bp was detected by transfecting UMR106 cells with plasmid consisting of *Furin*-VDR binding domain (Supplemental Table 2) fused to universal promoter (GGCAATCCGGTACTGTTGGTAAAGCCACC) and firefly luciferase (Figure 4C) (VectorBuilder). Construct sequences are shown in Supplemental Table 2. Cells were co-transfected with *Furin* promoter–luciferase reporter and pRL-TK (Renilla luciferase; Promega). Luciferase activity was measured by Dual-Luciferase Reporter Assay System (Promega). The firefly luciferase activity was normalized to Renilla. The experiments were performed in triplicates and were repeated 3 times.

### Human trial assessing the effects of ergocalciferol.

Details of the human trial have been previously published (46). We analyzed a subset of the samples from the randomized trial of ergocalciferol treatment in 18- to 45-year-old participants (*n* = 36) with 25-OHD levels ≤ 20 ng/mL (by chemiluminescence immunoassay). Participants were randomized to weekly ergocalciferol treatment of 50,000 IU (*n* = 18) or placebo (*n* = 18) for total of 12 weeks, while consuming a self-selected diet. Participant serum samples were frozen at –80°C. We selected all available samples from ergocalciferol-treated participants who had either stable or increased 1,25(OH)_2_D levels after 4 weeks of treatment and the same number of placebo-treated patients. Furin activity, iFGF23, cFGF23, 25-OHD, 1,25(OH)_2_D, PTH, serum phosphate, and serum calcium were measured at the initiation of the study and at 8 weeks of treatment.

### Human sample analysis.

Furin activity was measured by BioVision kit according to manufacturer’s instructions. cFGF23 was measured by Immutopics human ELISA kit. iFGF23, 25-OHD, 1,25(OH)_2_D, PTH, serum phosphate, and serum calcium were measured previously in the original study (46).

### Statistics.

We performed an unpaired, 2-tailed Student’s *t* test or 2-way ANOVA, depending on the number of groups analyzed, with significance defined as a *P* value of less than 0.05. Bonferroni’s correction was used for multiple comparisons by 2-tailed Student’s *t* test (that is, *P* values were multiplied by the number of comparisons).

### Study approval.

The human ergocalciferol treatment study sample analysis was approved by the IRB of the Massachusetts General Hospital and adhered to Declaration of Helsinki principles, and all participants provided written informed consent prior to inclusion in the study. Mouse studies were approved by the IACUC of Massachusetts General Hospital and were conducted under their guidelines.

### Data availability.

Data are available in the Supporting Data Values XLS file.

## Author contributions

HX, IB, WZ, ESL, and BA performed animal experiments, with input from HJ, EPR, MB, and PS. HX, IB, and ZC performed cellular experiments, with input from HJ, EPR, MB, and PS. HX, IB, WZ, BA, and IAPC performed serum ELISAs and furin activity measurements, with input from HJ, EPR, MB, and PS. SAMBB conducted the randomized clinical trial of assessing the effect of ergocalciferol treatment on FGF23. HJ, EPR, MB, and PS designed the experiments and analyzed results. PS, EPR, and MB wrote the manuscript with input from all authors.

## Supplementary Material

Supplemental data

Supporting data values

## Version 1 09/08/2023

Electronic publication
