# Supplementary material for: 1,25-Dihydroxyvitamin D3 regulates furin-mediated FGF23 cleavage
Source: JCI Insight. 2023 Sep 8;8(17):e168957. doi: 10.1172/jci.insight.168957 (PMC10544208; doi:10.1172/jci.insight.168957)
Supplement: Supplemental data [file jciinsight-8-168957-s049.pdf]

Supplemental Figures

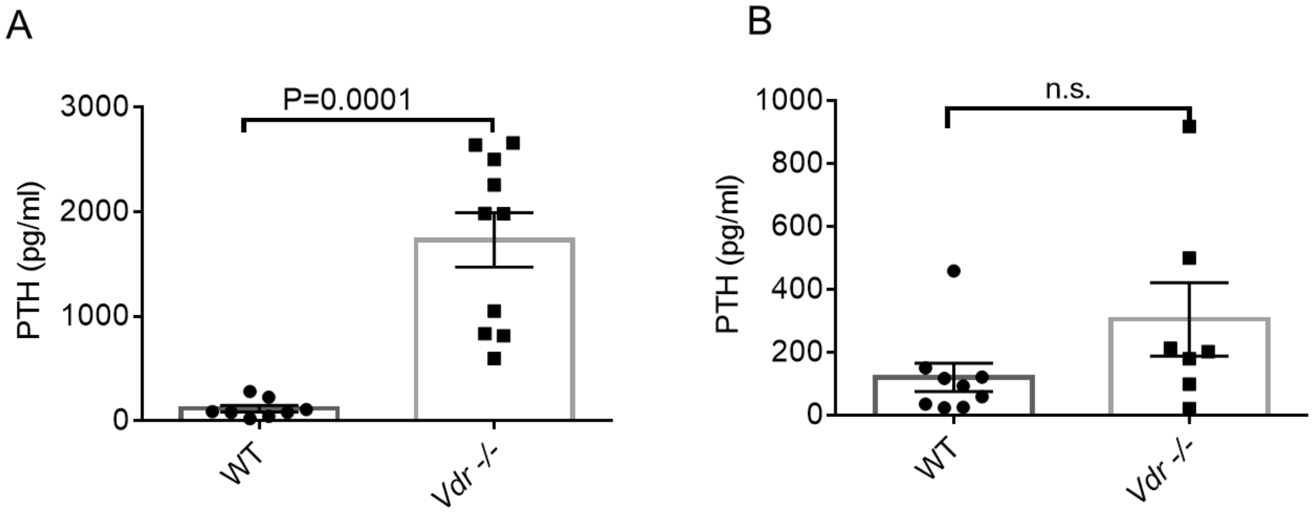

Supplemental Figure 1. The effect of *Vdr-/-* on PTH. Serum PTH is (**A**) increased in mice on regular diet (n=8-10 per group) and (**B**) there is no difference after 2 weeks of rescue diet (n=7-9 per group). Data represents the mean ± SEM. P values by two-tailed Student's t test.

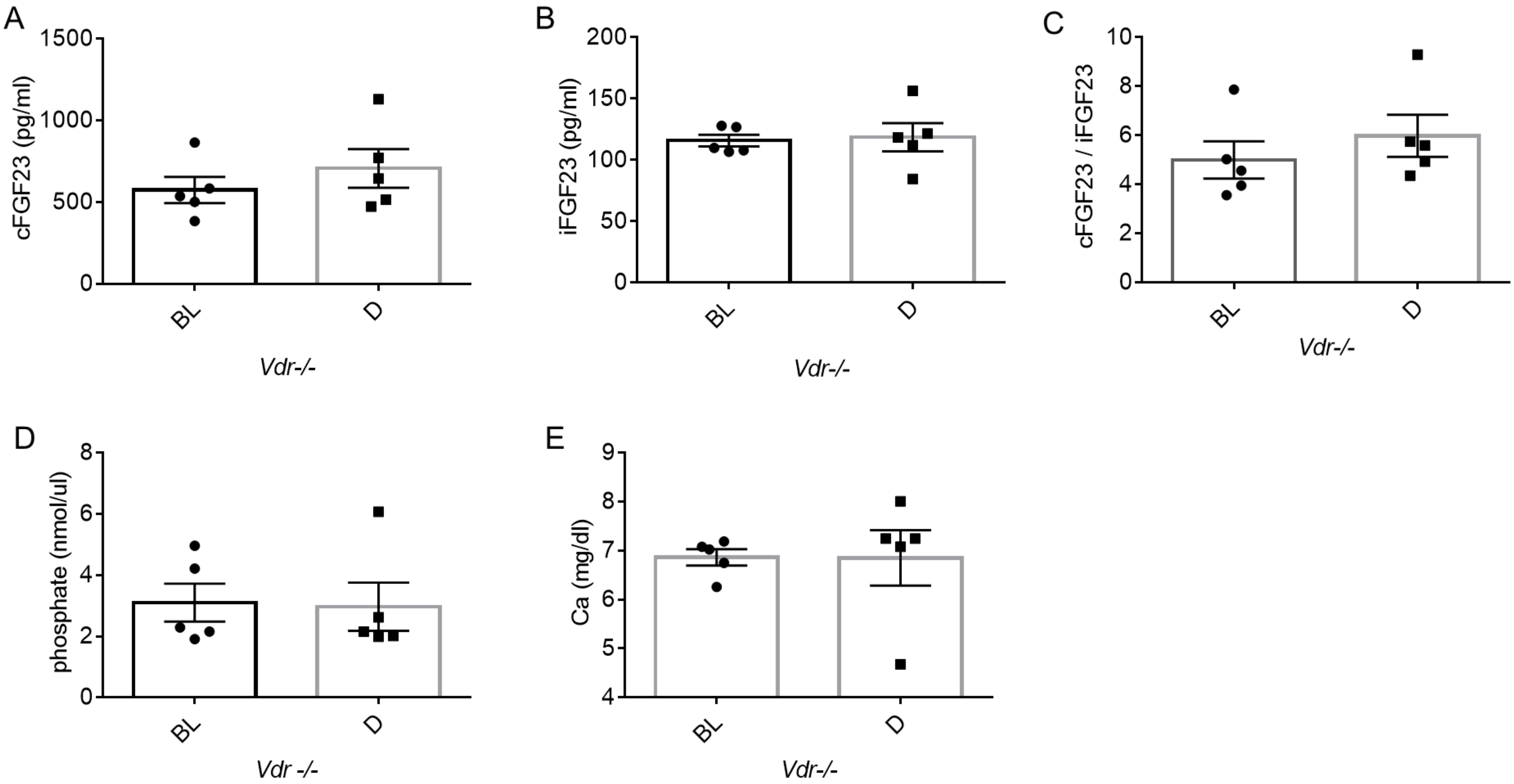

Supplemental Figure 2. 1,25(OH)$_2$D has no effect on FGF23 cleavage in *Vdr* -/- mice. *Vdr*-/- mice were treated with 1,25(OH)$_2$D (D) for 7 days have similar cFGF23 (**A**), iFGF23 (**B**), cFGF23/iFGF23 (**C**), phosphate (**D**) and Ca (**E**) levels as compared to baseline (n=5 per group). Data represents the mean ± SEM. Student's t test; D, 1,25(OH)$_2$D; BL, baseline

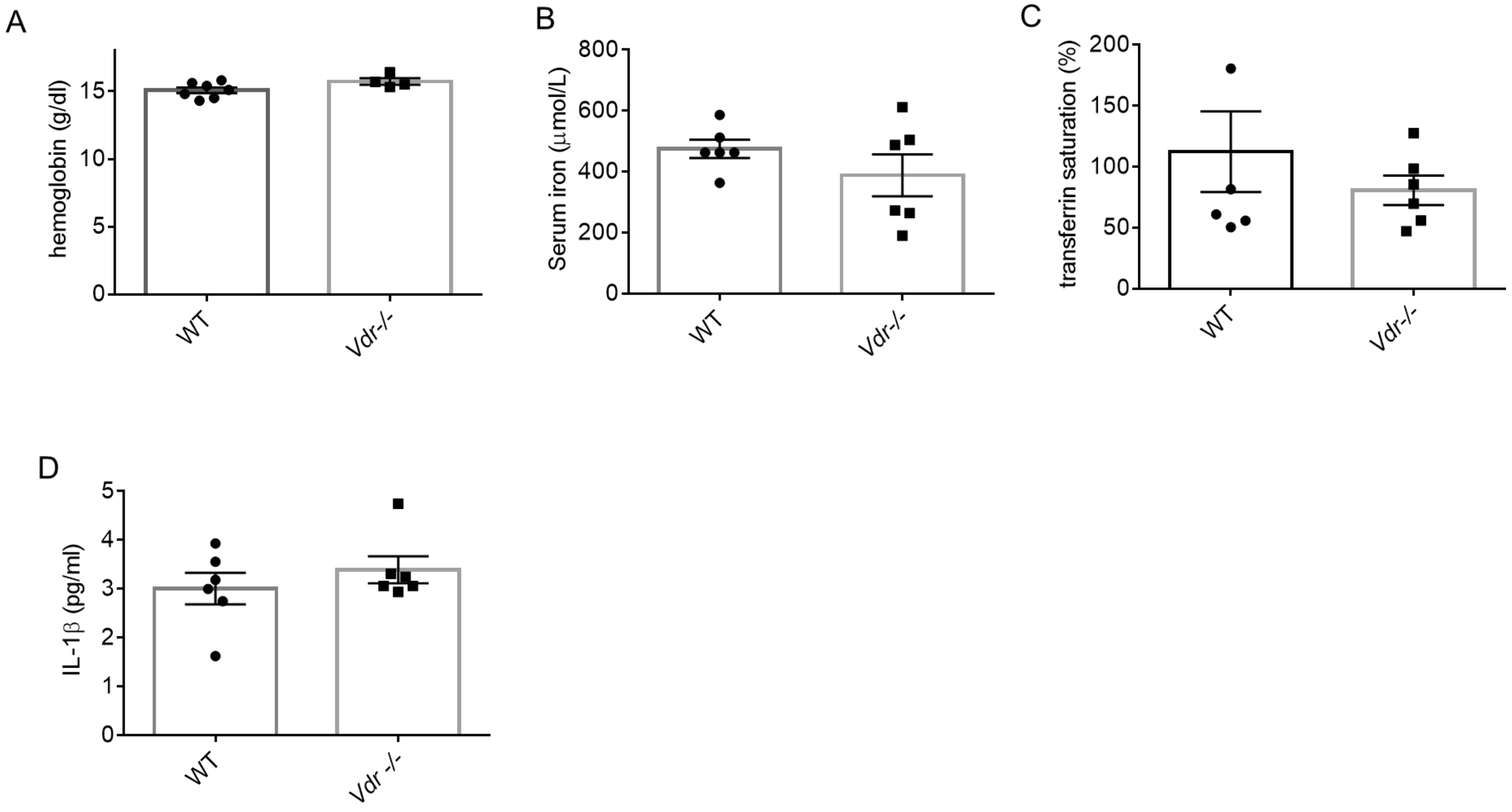

Supplemental Figure 3. *Vdr* -/- mice do not have anemia or inflammation. *Vdr*-/- mice have similar serum hemoglobin (n=4-7 per group) (**A**), iron (**B**), transferrin saturation (n=6 per group) (**C**) and IL-1β (n=6 per group) (**D**) levels as compared to wild-type (WT) mice. Data represents the mean ± SEM. Student's t test to determine whether significant changes were observed, WT, wild-type

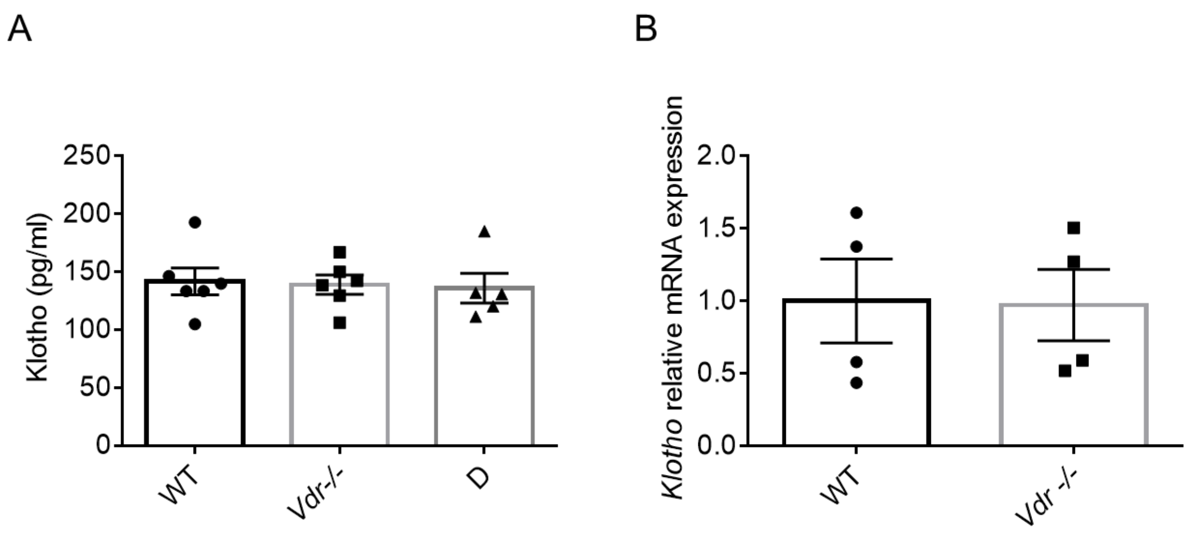

Supplemental Figure 4. *Vdr* -/- does not affect Klotho. *Vdr*-/- mice and 1,25$(OH)_2$D treated wild type mice have similar plasma Klotho levels as compared to WT mice (n=5-6 per group) (**A**). There is no difference in *Klotho* mRNA in the bone marrow of WT and *Vdr* -/- mice (n=4 per group) (**B**). Data represents the mean ± SEM. ANOVA and Student's t test to determine whether significant changes were observed, D, 1,25$(OH)_2$D; WT, wild type

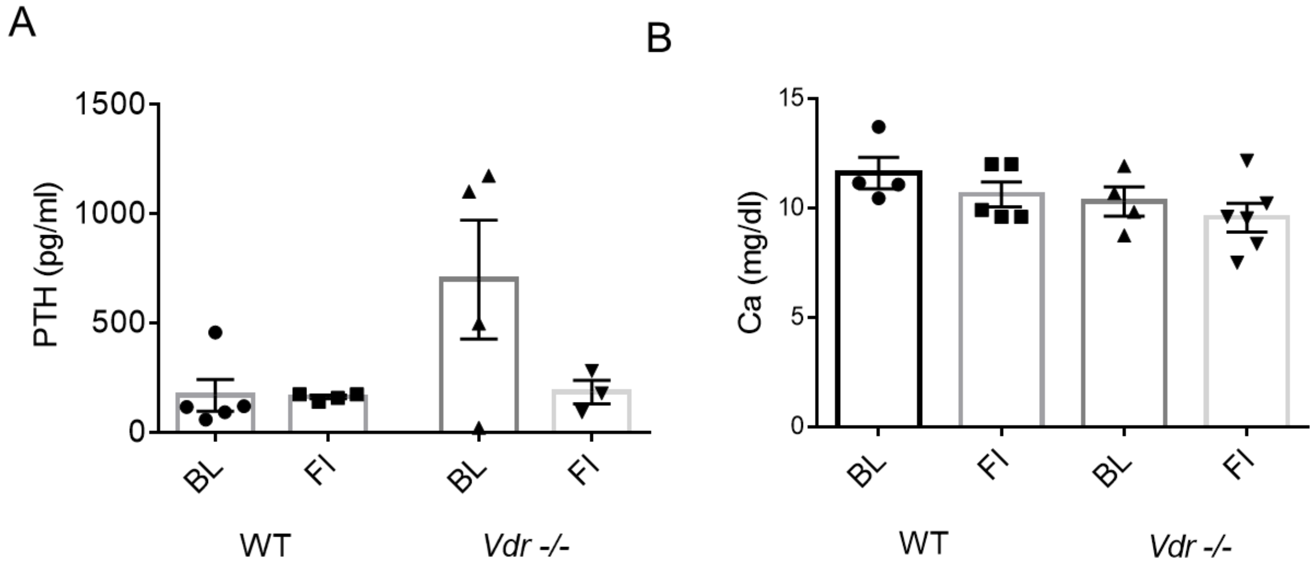

Supplemental Figure 5. Furin inhibitor treatment of WT and *Vdr-/-* mice. The effect on furin inhibitor treatment for 7 days on (**A**) serum PTH and (**B**) calcium levels (n=4-6 per group). Data represents the mean ± SEM. Non-significant, two way ANOVA. BL, baseline; FI, furin inhibitor; WT, wild-type

]

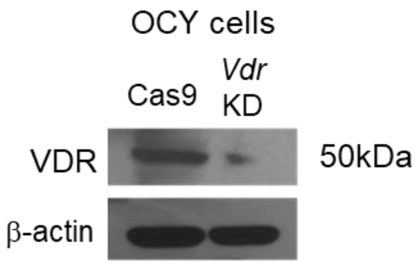

Supplemental Figure 6. Western blot of OCY454 cells in which the *Vdr* was deleted by CRISPR/Cas9. B–actin was used as a control.

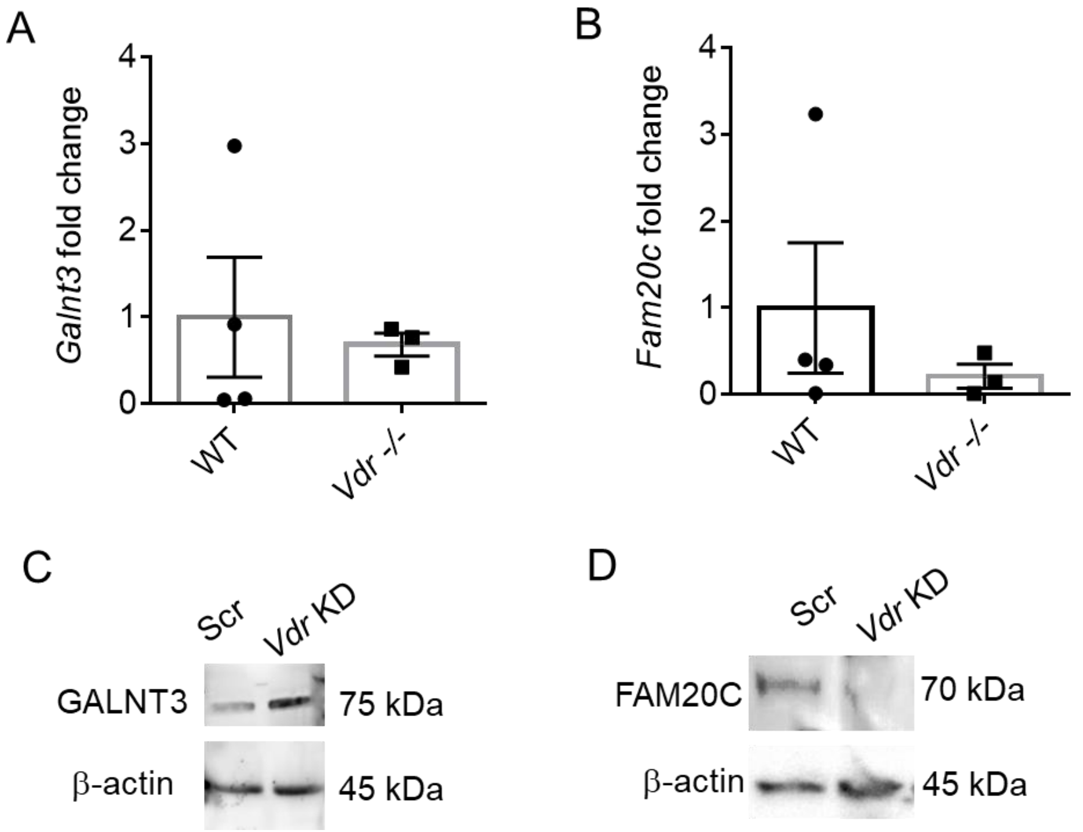

Supplemental Figure 7. *Galnt3* and *Fam20C* mRNA expression *in vivo* and GALNT3 and FAM20C protein *in vitro*. *GalnT3* **(A)** and *Fam20C* **(B)** mRNA expression in the bone marrow of WT and *Vdr* -/- mice (n=3-4 per group). Data represents the mean ± SEM, non-significant, two-tailed Student's t test.Western blot of GALNT3 (**C**) and FAM20C (**D**) from OCY454 cells. Scr, scramble, *Vdr* KD, *Vdr* knock-down

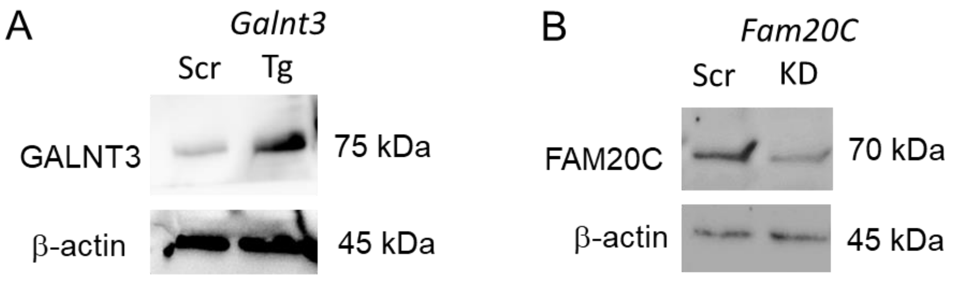

Supplemental Figure 8. *Galnt3* overexpression and *Fam20C* knock-down in OCY454 cells. GALNT3 (**A**) and FAM20C (**B**) Western blot after *Galnt3* overexpression (*Galnt3* Tg) and *Fam20C* knock-down (*Fam20C* KD).

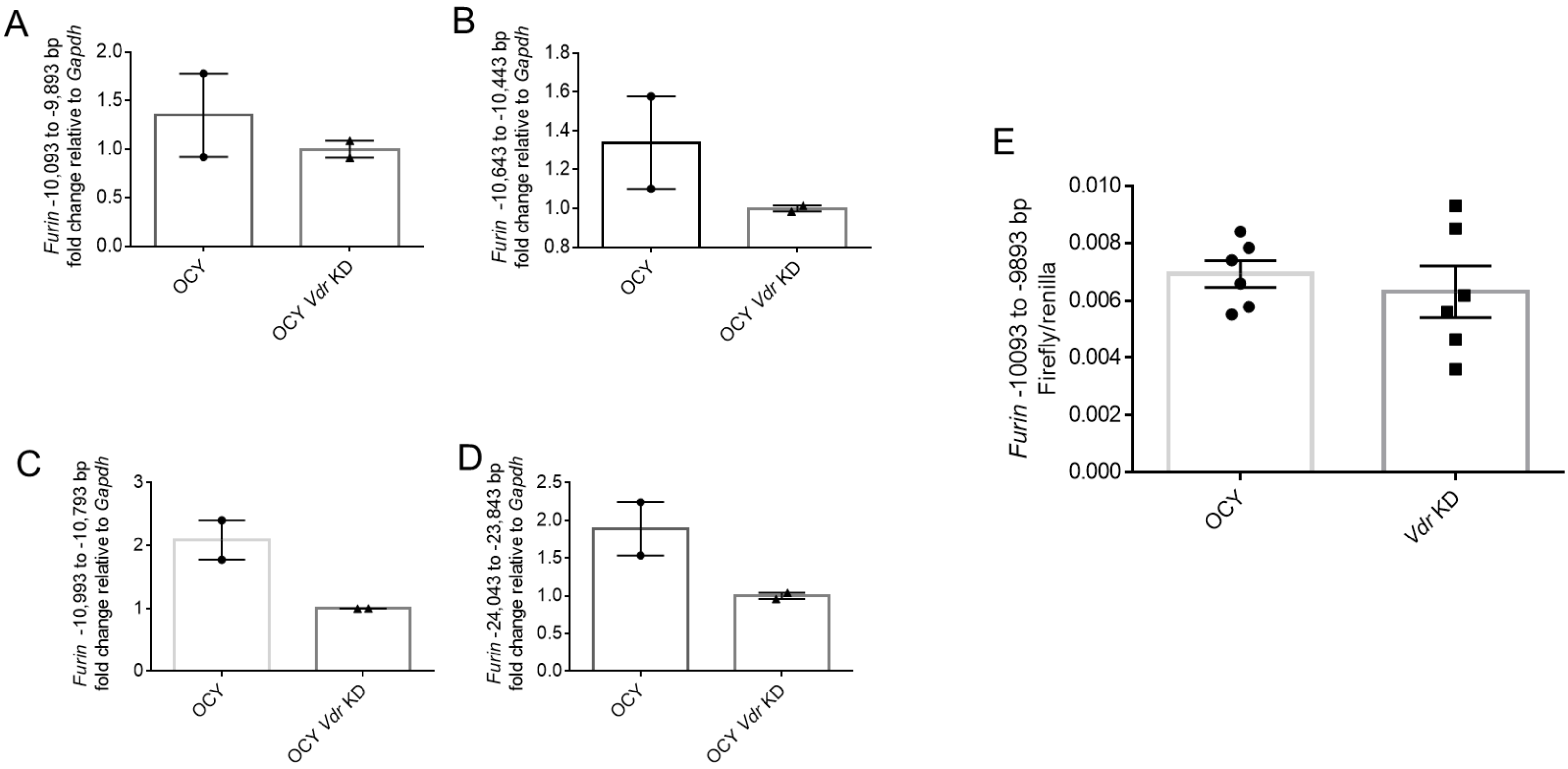

Supplemental Figure 9. *Furin* gene binding and transcription analyses. Chromatin Immunoprecipitation analysis of DNA regions identified by JASPR CORE transcription factor binding site program (**A-D**). (**E**) Firefly luciferase expression of control ChIP region -10093 to -9893 bp normalized to renilla luciferase (n=6 per group). Data represents the mean ± SEM, non-significant, two-tailed Student's t test.

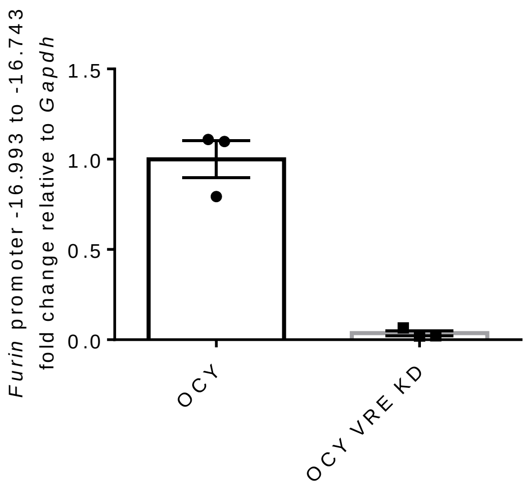

Supplemental Figure 10. qPCR showing reduction in transcription of *Furin* promoter -16,993 to -16,743 bp in *Vre* KD OCY454 cells. (n=3 per group)VRE KD, Vitamin D responsive element knock down

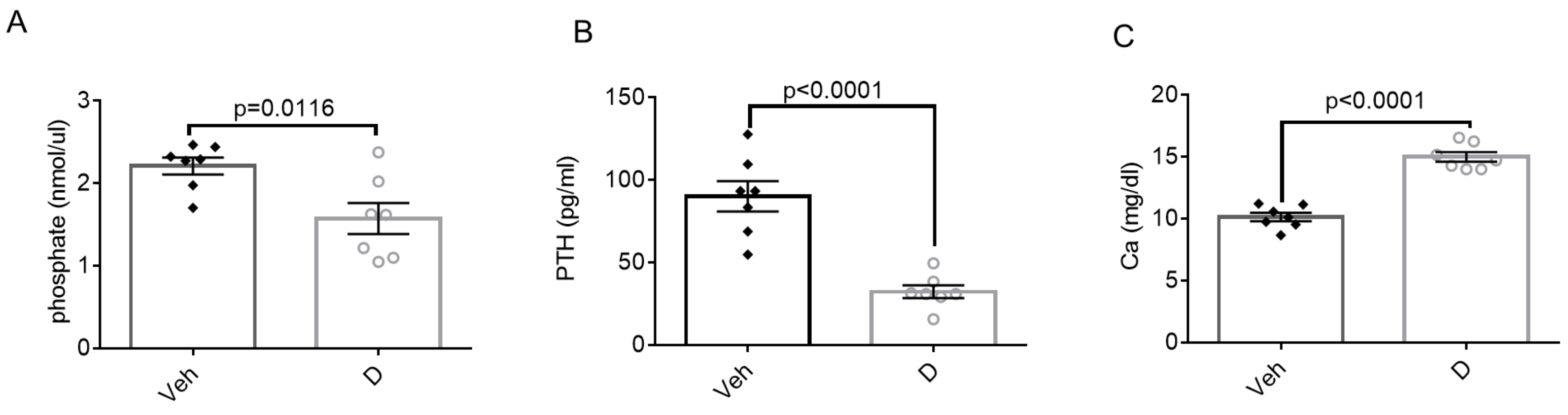

Supplemental Figure 11. The effect of 1,25(OH)$_2$D on serum bone mineral parameters. 1,25(OH)$_2$D 0.5 µg i.p. QOD for 7 days on serum (**A**) phosphate, (**B**) PTH and (**C**) calcium (n=7 per group). Data represents the mean ± SEM. P values by two-tailed Student's t test

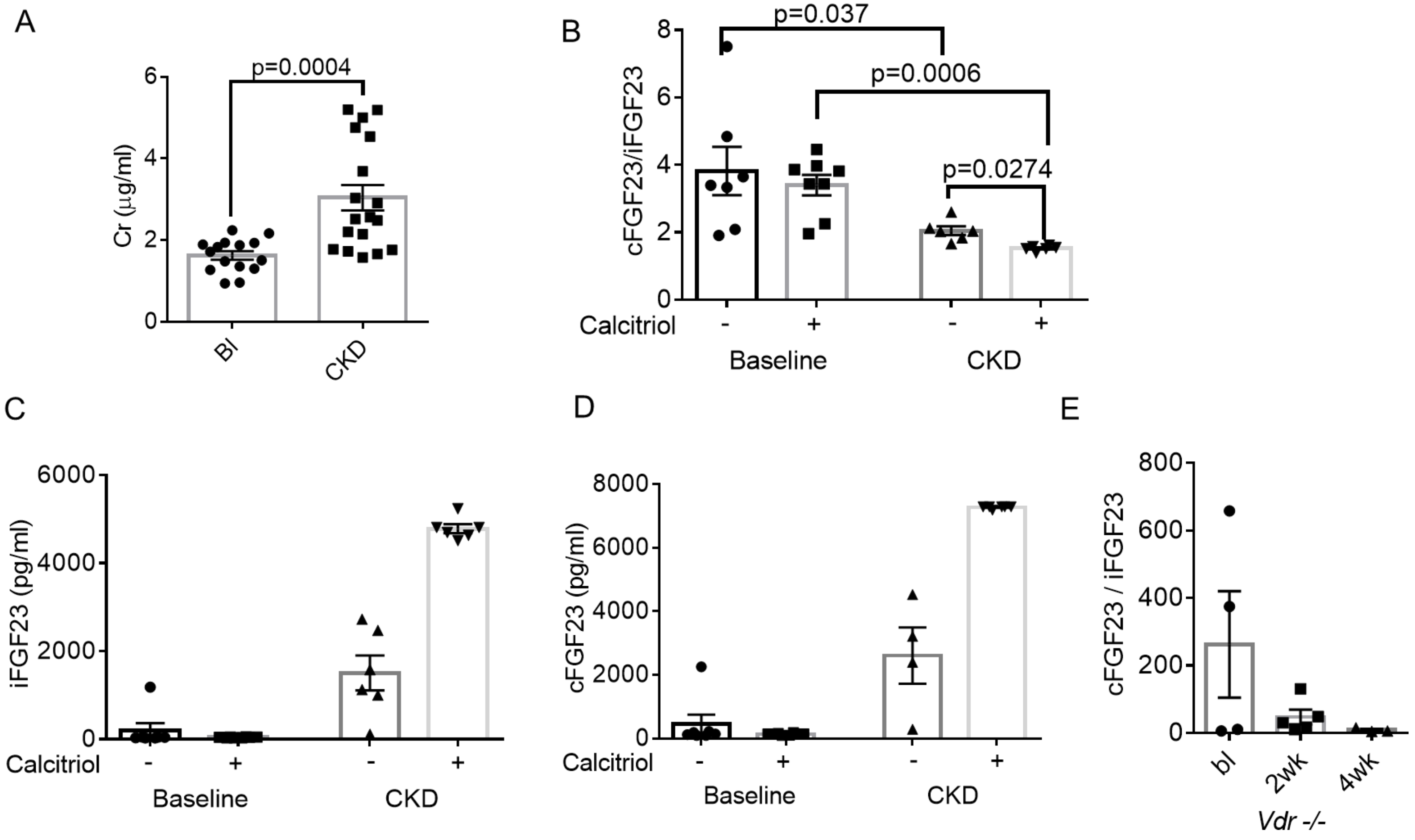

Supplemental Figure 12. The effect of calcitriol and *Vdr*-/- on FGF23 cleavage in CKD. (A) Creatinine is increased in mice on adenine diet induced CKD. (**B**) Adenine diet induced CKD reduces cFGF23/iFGF23 within 2 weeks as compared to baseline and calcitriol further reduces cFGF23/iFGF23. (**C**) iFGF23 and (**D**) cFGF23 are increased in CKD (n=6-8 per group). (**E**) *Vdr*-/- mice have reduction in cFGF23/iFGF23 with progression/weeks of CKD (n=3-5 per group). Data represents the mean ± SEM. P values by two way ANOVA. Cr, creatinine; Bl, baseline; CKD, chronic kidney diseas

Supplemental Table 1. Patient baseline characteristics

	PLACEBO (N=18)	ERGOCALCIFEROL (N=18)	*P* VALUE
**AGE**	30 (19, 44)	28 (18, 39)	0.39
**25OHD (ng/ml)**	15±4	15±3	0.93
**1.25(OH)$_2$D (pg/ml)**	36±13	37±11	0.83
**iFGF23 (pg/ml)**	47±34	41±17	0.50
**PTH (pg/ml)**	41±14	42±13	0.87
**Ca (mg/dl)**	9.0±0.4	9.2±0.4	0.12
**Phos (mg/dl)**	3.3±0.4	3.4±0.5	0.359
**Creatinine (mg/dl)**	0.9±0.1	0.9±0.1	0.67

Supplemental Table 2. List of oligonucleotides; all 5' to 3'

**RT-qPCR primer name**	**Primer sequence**
Furin FW	TGG TTG CTA TGG GTG GTC G
Furin RE	CCA GAA GTG GTA ATA GTC ACC GA
GalnT3 FW	TGC AAA TAG GAG CGC CCA TTA
GalnT3 RE	GGC GAT CAA AAA CCG GCT TC
Fam20C FW	GAT GTG ACG CGG GAT AAG AAG
Fam20C RE	GCT CGG TGG AAC AGT AGT AGG
Klotho FW	GAC GGT TTC GAG TGG CAT AGG
Klotho RE	CCG ACA CTG GGT TTT GTC AAA G
**sgRNA target name**	**sgRNA target sequence**
Vdr #1	TGGAGATTGCCGCATCACCA
Vdr #2	AGCGTTGAAGTGGAAGCCCG
VRE #1	TGCTTGCCTCTGCTTCCCAC
VRE #2	GTTTTTGTGTGTTTTTTTGC
**ChIP qPCR**	**ChIP qPCR primer sequence**
ChIP -10,093 to -9,893 bp FW	AGG CCC TGA GGT CTT AGC TA
ChIP -10,093 to -9,893 bp RE	CCT TCT CTG ACA CTC CAG AG
ChIP -10,643 to -10,443 bp FW	CCA TCG GAG GCT GAG CAT GG
ChIP -10,643 to -10,443 bp RE	CCA CAG TAG ACA GAT GGA GG
ChIP -10,993 to -10,793 bp FW	CAG ATG GAG AGG TTC TGT AC
ChIP -10,993 to -10,793 bp RE	ATG TAA GTA CAC TGT AGC TG
ChIP -16,993 to -16,743 bp FW	AAA ACA ACA CAA CAC AAC AA
ChIP -16,993 to -16,743 bp RE	TCC CAC ATG GTG GTG GAA AG
ChIP -24,043 to -23,843 bp FW	CCC AAG TGC TGG GAT TAA AG
ChIP -24,043 to -23,843 bp RE	ACA CCT TCC TTC CCC ACA CC

Supplemental Table 3. Plasmid DNA construct sequence by VectorBuilder Inc (Chicago, IL)

**Furin -16,993 to -16,743 and Firefly Construct**
CAACTTTGTATAGAAAAGTTGTCCCACATGGTGGTGGAAAGGAAGGCAGACACCAG<br>AGCCTTCTTTGCCTTGTGTAATTTTGAGAAAGTCTCATAGGCTAGCTTGGCCTCGAAC<br>TTCCTATGTAGCCTAGGGTGATCTTGAACTCCTGATCTTCCTGCCTCTCCCTCCTGAG<br>TGCTGGGATTATAGCATGTGCCACCATGTCTACCTTCCAGGCATTTGCTATTTGTGTT<br>TCTATATATATTTTGTTGTTGTTGTTGTGTTGTGTTGTTTTGGCAATCCGGTACTGTTG<br>GTAAAGCCACCCAAGTTTGTACAAAAAAGCAGGCTGCCACCATGGAAGACGCCAAA<br>AACATAAAGAAAGGCCCGGCGCCATTCTATCCGCTAGAGGATGGAACCGCTGGAGA<br>GCAACTGCATAAGGCTATGAAGAGATACGCCCTGGTTCCTGGAACAATTGCTTTTAC<br>AGATGCACATATCGAGGTGAACATCACGTACGCGGAATACTTCGAAATGTCCGTTCG<br>GTTGGCAGAAGCTATGAAACGATATGGGCTGAATACAAATCACAGAATCGTCGTAT<br>GCAGTGAAAACTCTCTTCAATTCTTTATGCCGGTGTTGGGCGCGTTATTTATCGGAGT<br>TGCAGTTGCGCCCGCGAACGACATTTATAATGAACGTGAATTGCTCAACAGTATGAA<br>CATTTCGCAGCCTACCGTAGTGTTTGTTTCCAAAAAGGGGTTGCAAAAAATTTTGAA<br>CGTGCAAAAAAAATTACCAATAATCCAGAAAATTATTATCATGGATTCTAAAACGG<br>ATTACCAGGGATTTCAGTCGATGTACACGTTCGTCACATCTCATCTACCTCCCGGTTT<br>TAATGAATACGATTTTGTACCAGAGTCCTTTGATCGTGACAAAACAATTGCACTGAT<br>AATGAACTCCTCTGGATCTACTGGGTTACCTAAGGGTGTGGCCCTTCCGCATAGAAC<br>TGCCTGCGTCAGATTCTCGCATGCCAGAGATCCTATTTTTGGCAATCAAATCATTCCG<br>GATACTGCGATTTTAAGTGTTGTTCCATTCCATCACGGTTTTGGAATGTTTACTACAC<br>TCGGATATTTGATATGTGGATTTCGAGTCGTCTTAATGTATAGATTTGAAGAAGAGC<br>TGTTTTTACGATCCCTTCAGGATTACAAAATTCAAAGTGCGTTGCTAGTACCAACCCT<br>ATTTTCATTCTTCGCCAAAAGCACTCTGATTGACAAATACGATTTATCTAATTTACAC<br>GAAATTGCTTCTGGGGGCGCACCTCTTTCGAAAGAAGTCGGGGAAGCGGTTGCAAA<br>ACGCTTCCATCTTCCAGGGATACGACAAGGATATGGGCTCACTGAGACTACATCAGC<br>TATTCTGATTACACCCGAGGGGGATGATAAACCGGGCGCGGTCGGTAAAGTTGTTCC<br>ATTTTTTGAAGCGAAGGTTGTGGATCTGGATACCGGGAAAACGCTGGGCGTTAATCA<br>GAGAGGCGAATTATGTGTCAGAGGACCTATGATTATGTCCGGTTATGTAAACAATCC<br>GGAAGCGACCAACGCCTTGATTGACAAGGATGGATGGCTACATTCTGGAGACATAG<br>CTTACTGGGACGAAGACGAACACTTCTTCATAGTTGACCGCTTGAAGTCTTTAATTA<br>AATACAAAGGATACCAGGTGGCCCCCGCTGAATTGGAGTCGATATTGTTACAACAC<br>CCCAACATCTTCGACGCGGGCGTGGCAGGTCTTCCCGACGATGACGCCGGTGAACTT<br>CCCGCCGCCGTTGTTGTTTTGGAGCACGGAAAGACGATGACGGAAAAAGAGATCGT<br>GGATTACGTCGCCAGTCAAGTAACAACCGCGAAAAAGTTGCGCGGAGGAGTTGTGT<br>TTGTGGACGAAGTACCGAAAGGTCTTACCGGAAAACTCGACGCAAGAAAAATCAGA<br>GAGATCCTCATAAAGGCCAAGAAGGGCGGAAAGTCCAAATTGTAAACCCAGCTTTC<br>TTGTACAAAGTGGTGATGGCCGGCCGCTTCGAGCAGACATGATAAGATACATTGAT<br>GAGTTTGGACAAACCACAACTAGAATGCAGTGAAAAAAATGCTTTATTTGTGAAATT<br>TGTGATGCTATTGCTTTATTTGTAACCATTATAAGCTGCAATAAACAAGTTAACAAC<br>AACAATTGCATTCATTTTATGTTTCAGGTTCAGGGGGAGGTGTGGGAGGTTTTTTAA<br>AGCAAGTAAAACCTCTACAAATGTGGTAGCGGCCGCGGCGCTCTTCCGCTTCCTCGC<br>TCACTGACTCGCTGCGCTCGGTCGTTCGGCTGCGGCGAGCGGTATCAGCTCACTCAA<br>AGGCGGTAATACGGTTATCCACAGAATCAGGGGATAACGCAGGAAAGAACATGTGA<br>GCAAAAGGCCAGCAAAAGGCCAGGAACCGTAAAAAGGCCGCGTTGCTGGCGTTTTT

CCATAGGCTCCGCCCCCCTGACGAGCATCACAAAAATCGACGCTCAAGTCAGAGGT
GGCGAAACCCGACAGGACTATAAAGATACCAGGCGTTTCCCCCTGGAAGCTCCCTC
GTGCGCTCTCCTGTTCCGACCCTGCCGCTTACCGGATACCTGTCCGCCTTTCTCTCTT
CGGGAAGCGTGGCGCTTTCTCATAGCTCACGCTGTAGGTATCTCAGTTCGGTGTAGG
TCGTTCGCTCCAAGCTGGGCTGTGTGCACGAACCCCCCGTTCAGCCCGACCGCTGCG
CCTTATCCGGTAACTATCGTCTTGAGTCCAACCCGGTAAGACACGACTTATCGCCAC
TGGCAGCAGCCACTGGTAACAGGATTAGCAGAGCGAGGTATGTAGGCGGTGCTACA
GAGTTCTTGAAGTGGTGGCCTAACTACGGCTACACTAGAAGAACAGTATTTGGTATC
TGCGCTCTGCTGAAGCCAGTTACCTTCGGAAAAAGAGTTGGTAGCTCTTGATCCGGC
AAACAAACCACCGCTGGTAGCGGTGGTTTTTTTGTTTGCAAGCAGCAGATTACGCGC
AGAAAAAAAGGATCTCAAGAAGATCCTTTGATCTTTTCTACGGGGTCTGACGCTCAG
TGGAACGAAAACTCACGTTAAGGGATTTTGGTCATGAGATTATCAAAAAGGATCTTC
ACCTAGATCCTTTTAAATTAAAAATGAAGTTTTAAATCAATCTAAAGTATATATGAG
TAAACTTGGTCTGACAGTTACCAATGCTTAATCAGTGAGGCACCTATCTCAGCGATC
TGTCTATTTCGTTCATCCATAGTTGCCTGACTCCCCGTCGTGTAGATAACTACGATAC
GGGAGGGCTTACCATCTGGCCCCAGTGCTGCAATGATACCGCGAGACCCACGCTCA
CCGGCTCCAGATTTATCAGCAATAAACCAGCCAGCCGGAAGGGCCGAGCGCAGAAG
TGGTCCTGCAACTTTATCCGCCTCCATCCAGTCTATTAATTGTTGCCGGGAAGCTAGA
GTAAGTAGTTCGCCAGTTAATAGTTTGCGCAACGTTGTTGCCATTGCTACAGGCATC
GTGGTGTCACGCTCGTCGTTTGGTATGGCTTCATTCAGCTCCGGTTCCCAACGATCAA
GGCGAGTTACATGATCCCCCATGTTGTGCAAAAAAGCGGTTAGCTCCTTCGGTCCTC
CGATCGTTGTCAGAAGTAAGTTGGCCGCAGTGTTATCACTCATGGTTATGGCAGCAC
TGCATAATTCTCTTACTGTCATGCCATCCGTAAGATGCTTTTCTGTGACTGGTGAGTA
CTCAACCAAGTCATTCTGAGAATAGTGTATGCGGCGACCGAGTTGCTCTTGCCCGGC
GTCAATACGGGATAATACCGCGCCACATAGCAGAACTTTAAAAGTGCTCATCATTGG
AAAACGTTCTTCGGGGCGAAAACTCTCAAGGATCTTACCGCTGTTGAGATCCAGTTC
GATGTAACCCACTCGTGCACCCAACTGATCTTCAGCATCTTTTACTTTCACCAGCGTT
TCTGGGTGAGCAAAAACAGGAAGGCAAAATGCCGCAAAAAAGGGAATAAGGGCGA
CACGGAAATGTTGAATACTCATACTCTTCCTTTTTCAATATTATTGAAGCATTTATCA
GGGTTATTGTCTCATGAGCGGATACATATTTGAATGTATTTAGAAAAATAAACAAAT
AGGGGTTCCGCGCACATTTCCCCGAAAAGTGCCACCTGACGTCTAAGAAACCATTAT
TATCATGACATTAACCTATAAAAATAGGCGTATCACGAGGCCCTTTCGTCGGCGCGC
CGCGGCCGC

Furin -10,093 to -9,893 Firefly Construct

CAACTTTGTATAGAAAAGTTGCCTTCTCTGACACTCCAGAGACAATGACTCTTCCCC
AAGGAGATCCACATCATAATGATGATGATGATGAAGATTTTGAGACAGGATCACAG
TAAAAAGCCCAGGCTAACTTTGAACTTAAAATCCTCTTGCCTCGGTTTCCCGAGTGC
TGGGATTGATTACAGGTGTGTACCACCATGCTAGCTAAGACCTCAGGGCCTGGCAAT
CCGGTACTGTTGGTAAAGCCACCCAAGTTTGTACAAAAAAGCAGGCTGCCACCATG
GAAGACGCCAAAAACATAAAGAAAGGCCCGGCGCCATTCTATCCGCTAGAGGATGG
AACCGCTGGAGAGCAACTGCATAAGGCTATGAAGAGATACGCCCTGGTTCCTGGAA
CAATTGCTTTTACAGATGCACATATCGAGGTGAACATCACGTACGCGGAATACTTCG
AAATGTCCGTTCGGTTGGCAGAAGCTATGAAACGATATGGGCTGAATACAAATCAC
AGAATCGTCGTATGCAGTGAAAACTCTCTTCAATTCTTTATGCCGGTGTTGGGCGCG
TTATTTATCGGAGTTGCAGTTGCGCCCGCGAACGACATTTATAATGAACGTGAATTG
CTCAACAGTATGAACATTTCGCAGCCTACCGTAGTGTTTGTTTCCAAAAAGGGGTTG

CAAAAAATTTTGAACGTGCAAAAAAAATTACCAATAATCCAGAAAATTATTATCAT
GGATTCTAAAACGGATTACCAGGGATTTCAGTCGATGTACACGTTCGTCACATCTCA
TCTACCTCCCGGTTTTAATGAATACGATTTTGTACCAGAGTCCTTTGATCGTGACAAA
ACAATTGCACTGATAATGAACTCCTCTGGATCTACTGGGTTACCTAAGGGTGTGGCC
CTTCCGCATAGAACTGCCTGCGTCAGATTCTCGCATGCCAGAGATCCTATTTTTGGC
AATCAAATCATTCCGGATACTGCGATTTTAAGTGTTGTTCCATTCCATCACGGTTTTG
GAATGTTTACTACACTCGGATATTTGATATGTGGATTTCGAGTCGTCTTAATGTATAG
ATTTGAAGAAGAGCTGTTTTTACGATCCCTTCAGGATTACAAAATTCAAAGTGCGTT
GCTAGTACCAACCCTATTTTCATTCTTCGCCAAAAGCACTCTGATTGACAAATACGA
TTTATCTAATTTACACGAAATTGCTTCTGGGGGCGCACCTCTTTCGAAAGAAGTCGG
GGAAGCGGTTGCAAAACGCTTCCATCTTCCAGGGATACGACAAGGATATGGGCTCA
CTGAGACTACATCAGCTATTCTGATTACACCCGAGGGGGATGATAAACCGGGCGCG
GTCGGTAAAGTTGTTCCATTTTTTGAAGCGAAGGTTGTGGATCTGGATACCGGGAAA
ACGCTGGGCGTTAATCAGAGAGGCGAATTATGTGTCAGAGGACCTATGATTATGTCC
GGTTATGTAAACAATCCGGAAGCGACCAACGCCTTGATTGACAAGGATGGATGGCT
ACATTCTGGAGACATAGCTTACTGGGACGAAGACGAACACTTCTTCATAGTTGACCG
CTTGAAGTCTTTAATTAAATACAAAGGATACCAGGTGGCCCCCGCTGAATTGGAGTC
GATATTGTTACAACACCCCAACATCTTCGACGCGGGCGTGGCAGGTCTTCCCGACGA
TGACGCCGGTGAACTTCCCGCCGCCGTTGTTGTTTTGGAGCACGGAAAGACGATGAC
GGAAAAAGAGATCGTGGATTACGTCGCCAGTCAAGTAACAACCGCGAAAAAGTTGC
GCGGAGGAGTTGTGTTTGTGGACGAAGTACCGAAAGGTCTTACCGGAAAACTCGAC
GCAAGAAAAATCAGAGAGATCCTCATAAAGGCCAAGAAGGGCGGAAAGTCCAAAT
TGTAAACCCAGCTTTCTTGTACAAAGTGGTGATGGCCGGCCGCTTCGAGCAGACATG
ATAAGATACATTGATGAGTTTGGACAAACCACAACTAGAATGCAGTGAAAAAAATG
CTTTATTTGTGAAATTTGTGATGCTATTGCTTTATTTGTAACCATTATAAGCTGCAAT
AAACAAGTTAACAACAACAATTGCATTCATTTTATGTTTCAGGTTCAGGGGGAGGTG
TGGGAGGTTTTTTAAAGCAAGTAAAACCTCTACAAATGTGGTAGCGGCCGCGGCGCT
CTTCCGCTTCCTCGCTCACTGACTCGCTGCGCTCGGTCGTTCGGCTGCGGCGAGCGG
TATCAGCTCACTCAAAGGCGGTAATACGGTTATCCACAGAATCAGGGGATAACGCA
GGAAAGAACATGTGAGCAAAAGGCCAGCAAAAGGCCAGGAACCGTAAAAAGGCCG
CGTTGCTGGCGTTTTTCCATAGGCTCCGCCCCCCTGACGAGCATCACAAAAATCGAC
GCTCAAGTCAGAGGTGGCGAAACCCGACAGGACTATAAAGATACCAGGCGTTTCCC
CCTGGAAGCTCCCTCGTGCGCTCTCCTGTTCCGACCCTGCCGCTTACCGGATACCTGT
CCGCCTTTCTCTCTTCGGGAAGCGTGGCGCTTTCTCATAGCTCACGCTGTAGGTATCT
CAGTTCGGTGTAGGTCGTTCGCTCCAAGCTGGGCTGTGTGCACGAACCCCCCGTTCA
GCCCGACCGCTGCGCCTTATCCGGTAACTATCGTCTTGAGTCCAACCCGGTAAGACA
CGACTTATCGCCACTGGCAGCAGCCACTGGTAACAGGATTAGCAGAGCGAGGTATG
TAGGCGGTGCTACAGAGTTCTTGAAGTGGTGGCCTAACTACGGCTACACTAGAAGA
ACAGTATTTGGTATCTGCGCTCTGCTGAAGCCAGTTACCTTCGGAAAAAGAGTTGGT
AGCTCTTGATCCGGCAAACAAACCACCGCTGGTAGCGGTGGTTTTTTTGTTTGCAAG
CAGCAGATTACGCGCAGAAAAAAAGGATCTCAAGAAGATCCTTTGATCTTTTCTACG
GGGTCTGACGCTCAGTGGAACGAAAACTCACGTTAAGGGATTTTGGTCATGAGATTA
TCAAAAAGGATCTTCACCTAGATCCTTTTAAATTAAAAATGAAGTTTTAAATCAATC
TAAAGTATATATGAGTAAACTTGGTCTGACAGTTACCAATGCTTAATCAGTGAGGCA
CCTATCTCAGCGATCTGTCTATTTCGTTCATCCATAGTTGCCTGACTCCCCGTCGTGT
AGATAACTACGATACGGGAGGGCTTACCATCTGGCCCCAGTGCTGCAATGATACCG

CGAGACCCACGCTCACCGGCTCCAGATTTATCAGCAATAAACCAGCCAGCCGGAAG
GGCCGAGCGCAGAAGTGGTCCTGCAACTTTATCCGCCTCCATCCAGTCTATTAATTG
TTGCCGGGAAGCTAGAGTAAGTAGTTCGCCAGTTAATAGTTTGCGCAACGTTGTTGC
CATTGCTACAGGCATCGTGGTGTCACGCTCGTCGTTTGGTATGGCTTCATTCAGCTCC
GGTTCCCAACGATCAAGGCGAGTTACATGATCCCCCATGTTGTGCAAAAAAGCGGTT
AGCTCCTTCGGTCCTCCGATCGTTGTCAGAAGTAAGTTGGCCGCAGTGTTATCACTC
ATGGTTATGGCAGCACTGCATAATTCTCTTACTGTCATGCCATCCGTAAGATGCTTTT
CTGTGACTGGTGAGTACTCAACCAAGTCATTCTGAGAATAGTGTATGCGGCGACCGA
GTTGCTCTTGCCCGGCGTCAATACGGGATAATACCGCGCCACATAGCAGAACTTTAA
AAGTGCTCATCATTGGAAAACGTTCTTCGGGGCGAAAACTCTCAAGGATCTTACCGC
TGTTGAGATCCAGTTCGATGTAACCCACTCGTGCACCCAACTGATCTTCAGCATCTTT
TACTTTCACCAGCGTTTCTGGGTGAGCAAAAACAGGAAGGCAAAATGCCGCAAAAA
AGGGAATAAGGGCGACACGGAAATGTTGAATACTCATACTCTTCCTTTTTCAATATT
ATTGAAGCATTTATCAGGGTTATTGTCTCATGAGCGGATACATATTTGAATGTATTTA
GAAAAATAAACAAATAGGGGTTCCGCGCACATTTCCCCGAAAAGTGCCACCTGACG
TCTAAGAAACCATTATTATCATGACATTAACCTATAAAAATAGGCGTATCACGAGGC
CCTTTCGTCGGCGCGCCGCGGCCGC
